# Identification of lysosomal lipolysis as an essential noncanonical mediator of adipocyte fasting and cold-induced lipolysis

**DOI:** 10.1172/JCI185340

**Published:** 2025-03-17

**Authors:** Yu-Sheng Yeh, Trent D. Evans, Mari Iwase, Se-Jin Jeong, Xiangyu Zhang, Ziyang Liu, Arick Park, Ali Ghasemian, Borna Dianati, Ali Javaheri, Dagmar Kratky, Satoko Kawarasaki, Tsuyoshi Goto, Hanrui Zhang, Partha Dutta, Francisco J. Schopfer, Adam C. Straub, Jaehyung Cho, Irfan J. Lodhi, Babak Razani

**Affiliations:** 1 Department of Medicine and Vascular Medicine Institute, University of Pittsburgh School of Medicine and University of Pittsburgh Medical Center (UPMC), Pittsburgh, Pennsylvania, USA.; 2 Pittsburgh VA Medical Center, Pittsburgh, Pennsylvania, USA.; 3 Cardiovascular Division, Washington University School of Medicine, St. Louis, Missouri, USA.; 4 Division of Oncology, Washington University School of Medicine, St. Louis, Missouri, USA.; 5 John Cochran VA Medical Center, St. Louis, Missouri, USA.; 6 Gottfried Schatz Research Center for Cell Signaling, Metabolism and Aging, Division of Molecular Biology and Biochemistry, Medical University of Graz, Graz, Austria.; 7 Division of Food Science and Biotechnology, Graduate School of Agriculture, Kyoto University, Kyoto, Japan.; 8 Research Unit for Physiological Chemistry, Center for the Promotion of Interdisciplinary Education and Research, Kyoto University, Kyoto, Japan.; 9 Department of Medicine, Columbia University Irving Medical Center, New York, New York, USA.; 10 Department of Pharmacology and Chemical Biology, University of Pittsburgh School of Medicine and UPMC, Pittsburgh, Pennsylvania, USA.; 11 Division of Hematology, Washington University School of Medicine, St. Louis, Missouri, USA.; 12 Department of Pathology and Immunology, Washington University School of Medicine, St. Louis, Missouri, USA.; 13 Division of Endocrinology, Metabolism, and Lipid Research, Department of Medicine, Washington University School of Medicine, St. Louis, Missouri, USA.

**Keywords:** Endocrinology, Metabolism, Therapeutics, Adipose tissue, Lysosomes, Obesity

## Abstract

Adipose tissue lipolysis is the process by which triglycerides in lipid stores are hydrolyzed into free fatty acids (FFAs), serving as fuel during fasting or cold-induced thermogenesis. Although cytosolic lipases are considered the predominant mechanism of liberating FFAs, lipolysis also occurs in lysosomes via lysosomal acid lipase (LIPA), albeit with unclear roles in lipid storage and whole-body metabolism. We found that adipocyte LIPA expression increased in adipose tissue of mice when lipolysis was stimulated during fasting, cold exposure, or β-adrenergic agonism. This was functionally important, as inhibition of LIPA genetically or pharmacologically resulted in lower plasma FFAs under lipolytic conditions. Furthermore, adipocyte LIPA deficiency impaired thermogenesis and oxygen consumption and rendered mice susceptible to diet-induced obesity. Importantly, lysosomal lipolysis was independent of adipose triglyceride lipase, the rate-limiting enzyme of cytosolic lipolysis. Our data suggest a significant role for LIPA and lysosomal lipolysis in adipocyte lipid metabolism beyond classical cytosolic lipolysis.

## Abstract 2

We describe an alternative route for the release of lipids from adipocytes which utilizes lysosomes and which has importance in the development of obesity and metabolic disease.

## Graphical abstract

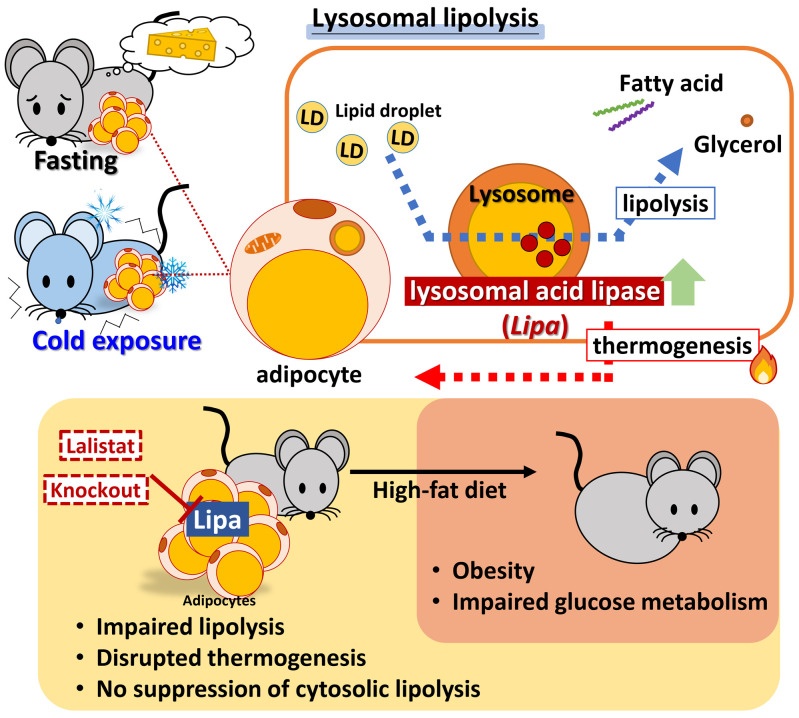

## Introduction

A defining feature of obesity is the increased storage of energy as triglycerides (TGs) within adipocytes. The study of the mechanisms leading to the net accumulation of TGs is paramount to understanding how both obesity and associated metabolic consequences such as type 2 diabetes develop (1). During postprandial conditions, glycerol-3-phosphate is esterified with lipoprotein lipase-derived free fatty acids (FFAs) to form TGs and build the adipocyte lipid droplet (2). Conversely, physiological conditions such as aerobic exercise, cold exposure (CE), and fasting demand the liberation of FFAs from TGs via lipolysis for use as energy substrates (3). This occurs both locally, wherein FFAs are oxidized directly in mitochondria, and peripherally, wherein white adipose tissue (WAT) lipolysis generates FFAs for release in the circulation and use in liver, skeletal muscle, and brown adipose mitochondria.

A great deal of effort has defined the set of reactions governing adipocyte lipolysis. At the center of these lies the stepwise removal of each FFA by adipose triglyceride lipase (ATGL/PNPLA2), hormone sensitive lipase (HSL/LIPE), and monoglyceride lipase (MGL/MGLL) (4). Activities of these enzymes titrate lipolytic flux to meet physiological energy demands. For example, the expression of ATGL is increased during fasting (5) and suppressed with insulin (6). Further, posttranscriptional regulation heavily governs ATGL activity, wherein comparative gene identification-58 (CGI-58/ABHD5) acts as an activator and G0/G1 switch gene 2 (G0S2) as an inhibitor (7, 8). Disruption in many of these nodes amounts to expected physiological consequences, such as defective FFA generation for thermogenesis in adipocyte-specific ATGL deficiency (9) and an increased frequency of large adipocyte droplets with adipocyte-specific ATGL or HSL deficiency (9, 10). Outside of these central roles in healthy energy metabolism, dysregulation of lipolysis is a key feature in obesity and the progression to more overt disease. Fatty acids are fairly inert when stored as TGs in lipid droplets, but these and derived species such as ceramides may be toxic at higher concentrations and are potentially responsible for many of the pathologies associated with cooccurring metabolic disorders such as type II diabetes and nonalcoholic steatohepatitis. Additionally, insulin resistance in adipocytes results in a failure to suppress lipolysis with adverse lipid spillover into other tissues (11). Despite the clear importance of the ATGL/HSL/MGL cytosolic lipolysis pathway to total adipocyte lipolysis, several lines of evidence point toward the existence of yet-to-be-defined alternative and complementary pathways. For example, TG hydrolysis is still observed in the absence of ATGL (9, 12) or HSL (13). Defining these remaining pathways and how they might interact with cytosolic lipolysis is an ongoing and crucial task in lipolysis research.

One such alternative pathway is lysosomal lipolysis mediated by the key pH-sensitive TG and cholesterol-ester hydrolase lysosomal acid lipase (LIPA). Lipids can be delivered to the lysosome and LIPA either through endocytic routes or a relatively recently described autophagic route termed lipophagy. Early studies defined the relevance of this pathway in the delivery of either lipid droplet–derived TGs or cholesterol esters to the lysosome in hepatocytes (14) or atherosclerotic macrophages (15). However, whether lysosomal/LIPA-mediated TG lipolysis is relevant and functions independently of cytosolic lipolysis in adipocytes has only been partially evaluated. One study demonstrated cytosolic lipolysis produces small lipid droplets that are amenable for lysosomal lipolysis (16, 17). Additionally, mice with whole-body LIPA deficiency demonstrate defective thermogenesis and lipid accumulation in brown adipose tissue (BAT) (18), though the roles of LIPA in other tissues affected by the whole-body knockout model complicate the interpretation of these phenotypes (19). In the present study, we sought to test the role of LIPA as an overall contributor to adipocyte lipolysis and key downstream physiological outcomes such as nonshivering thermogenesis. Using a combination of in vitro adipocyte lipolysis models and murine models of genetic or pharmacologically-induced LIPA deficiency, we define an essential role for lysosomal lipolysis in adipocytes.

## Results

### LIPA in adipocytes is stimulated with fasting and CE.

**Figure 1:**
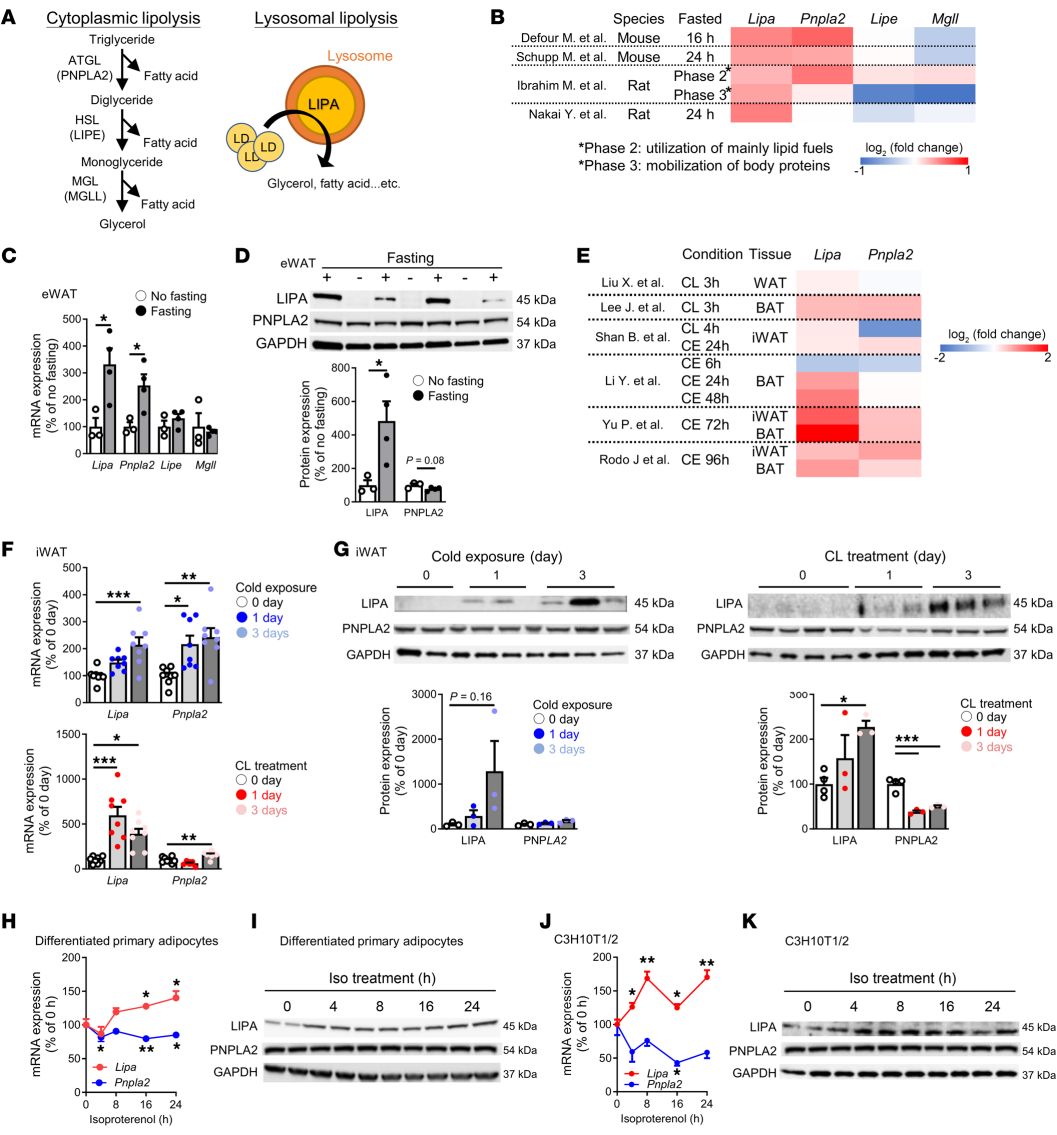
LIPA in adipocytes is stimulated by fasting, CE, and β-agonists. (**A**) Schematic illustration of cytosolic versus lysosomal lipolysis and key enzymes involved. (**B**) Experimental information and log_2_ fold changes of lipase gene expression in datasets (60–63) characterizing eWAT during fasting. (**C**) Gene expression of Lipa and cytosolic lipases and (**D**) protein expression of LIPA and ATGL (PNPLA2) in eWAT from mice fasted 16 hours (*n* = 3–4). (**E**) log_2_ Fold changes of Lipa and ATGL (Pnpla2) gene expression in datasets (64–68) examining CE or CL treatment. (**F**) Gene expression of Lipa and ATGL (Pnpla2) (*n* = 8) and (**G**) protein expression of LIPA and ATGL (PNPLA2) (*n* = 3–4) in iWAT from mice housed at 4ºC (left panel) or treated with CL (right panel) for 1 or 3 days. (**H**) Gene expression of *Lipa* and ATGL (*Pnpla2*) (*n* = 5–6) and (**I**) protein expression of LIPA and ATGL (PNPLA2) in differentiated primary adipocytes derived from iWAT SV cells treated with isoproterenol for indicted durations. (**J**) Gene expression of *Lipa* and ATGL (*Pnpla2*) (*n* = 4) and (**K**) protein expression of LIPA and ATGL (PNPLA2) in C3H1T1/2 adipocytes treated with isoproterenol for indicated durations. All mice were male and fed an ND. Values are presented as mean ± SEM. Significant differences were determined by Student’s *t* test (**C**, **D**, **H**, and **J**) or 1-way ANOVA with a post hoc Tukey’s HSD (**F** and **G**) for comparisons with the indicated groups. *P < 0.05; **P < 0.01; ***P < 0.001. See also associated Supplemental Figures 1–4.

To understand the role of lysosomal lipolysis in adipocyte TG metabolism and how it is regulated compared with the established ATGL/HSL/MGL cytosolic lipolysis pathway (Figure 1A), we first examined available datasets profiling rodent WAT gene expression in response to fasting, a canonical stimulus requiring lipolysis to meet energy demands. Although many posttranslational mechanisms regulate the activity of ATGL and HSL (3), only ATGL (*Pnpla2*) expression was increased in the majority (3/5) of fasting studies analyzed. HSL (*Lipe*) and MGL (*Mgll*) expression were unchanged or even downregulated in some cases. In contrast, expression of LIPA (*Lipa*), the only enzyme mediating lysosomal lipolysis, was robustly upregulated in all models (Figure 1B), suggesting a role in adipose lipolysis that may be comparatively impactful during longer fasting.

To extend these findings and further establish increased LIPA expression as part of the fasting response, we examined epididymal WAT (eWAT) LIPA (*Lipa*) expression in mice induced by 16 hours of fasting-induced lipolysis (Supplemental Figure 1; supplemental material available online with this article; https://doi.org/10.1172/JCI185340DS1) and observed increases in both gene and protein expression (Figure 1, C and D). This was recapitulated in both nutrient-depleted cultured primary adipocytes (i.e., differentiated inguinal WAT–derived [iWAT-derived] stromal vascular [SV] cells [Supplemental Figure 2, A and B]) as well as the C3H10T1/2 adipocyte cell line (Supplemental Figure 2, C and D). The expression of housekeeping gene 36B4 (*Rplp0*) remained unchanged in both cell types (Supplemental Figure 2, E and F). In contrast, ATGL (*Pnpla2*) gene expression, but not that of HSL (*Lipe*) and MGL (*Mgll*), was upregulated in the eWAT of mice fasted 16 hours (Figure 1C), and ATGL (PNPLA2) protein expression was unchanged (Figure 1D). Similarly, minimal or less robust changes were observed in ATGL (*Pnpla2*) expression in nutrient-depleted primary adipocytes and C3H10T1/2 cells (Supplemental Figure 2, A and C). Overall, these data demonstrate that increased LIPA expression is a prominent and readily observed feature of the adipose transcriptional-translational response to fasting compared with the cytosolic lipases.

A second physiological stimulus well known to induce lipolysis through the β-adrenergic pathway is CE. We next analyzed publicly available gene expression datasets profiling BAT or WAT under the conditions of CE or administration of CL316,243 (CL), a β3-adrenergic receptor agonist, to see whether *Lipa* expression was also associated with either condition. Both CE and CL consistently stimulated *Lipa* expression in WAT or BAT overall, and showed *Lipa* expression is upregulated during longer, but not short-term CE (Figure 1E). To dissect the possible time dependence of LIPA expression in this context, we housed C57BL/6J mice at 4°C or 24°C for either 1 or 3 days. Consistent with prior reports (20, 21), longer-term CE and CL reduced steady-state levels of plasma FFAs and TGs (Supplemental Figure 3A), a reduction thought to be due to high dependence on the net consumption of FFAs by β-oxidation in these prolonged scenarios. Both conditions also increased gene and protein expression of LIPA in iWAT (Figure 1, F and G) and BAT (Supplemental Figure 3, B and C), whereas induction of ATGL (*Pnpla2*) was overall more modest and less consistent (Figure 1, F and G, and Supplemental Figure 3, B, and C). In addition, LIPA gene and protein levels were increased with the administration of isoproterenol, a β-adrenergic agonist, in cultured primary adipocytes (Figure 1, H and I) and in C3H10T1/2 cells (Figure 1, J and K) while the increase in ATGL (*Pnpla2*) was minimal. Taken together, these data demonstrate a strong and consistent association of increased LIPA expression with classic physiological scenarios demanding lipolysis.

### Macrophage depletion does not affect adipose LIPA expression under lipolytic conditions.

Given that monocytes and macrophages express lysosomal genes including LIPA and are recruited into adipose tissue in response to metabolic stressors such as cold and obesity (22–24), we evaluated the contribution of macrophages to adipose tissue LIPA expression with fasting or CE. Clodronate liposomes were used to deplete monocytes and macrophages (25). As shown in Supplemental Figure 4A, macrophage markers ITGAM (CD11b) and ADGRE1 (F4/80) were diminished in the eWAT of high-fat diet–induced (HFD-induced) obese mice following clodronate treatment. The same strategy was applied to confirm the effect of monocyte/macrophage depletion on adipose LIPA expression stimulated by fasting, CE, and CL treatment (Supplemental Figure 4B). Fasting-induced gene and protein expression of LIPA in eWAT were not affected by clodronate treatment (Supplemental Figure 4, C and D). Similarly, clodronate treatment had no effect on increased LIPA expression in iWAT induced by 3 days of CE or CL treatment (Supplemental Figure 4, E and F). This evidence suggests that monocytes and macrophages play a negligible role in regulating LIPA expression in adipose tissue during lipolytic stress.

### Adipocyte LIPA deficiency does not affect plasma FFA and TG levels and baseline metabolism.

**Figure 2:**
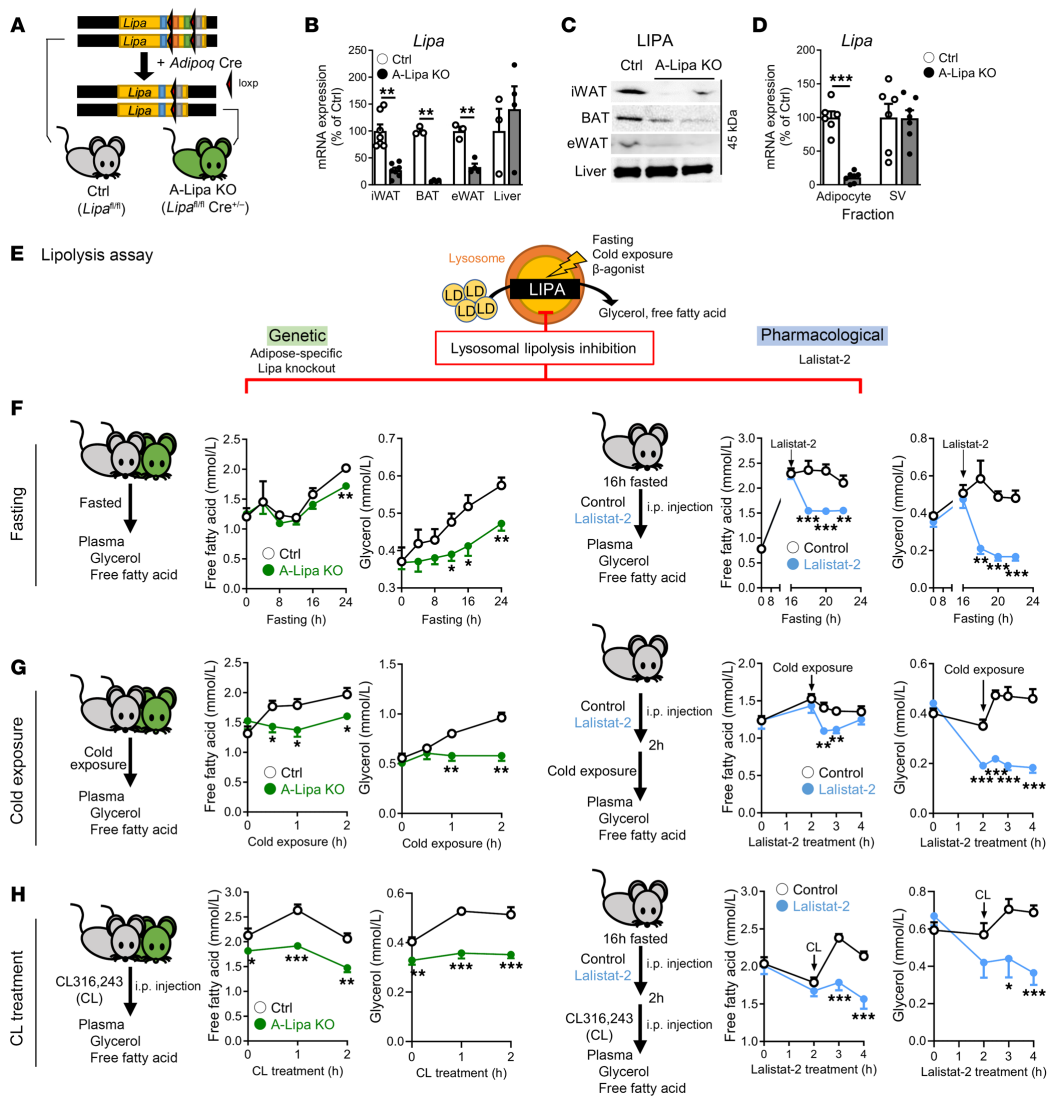
LIPA disruption suppresses fasting-, cold-, and β-agonist-induced lipolysis in mouse models. (**A**) Experimental strategy outlining *Adipoq*-Cre–driven knockout of LIPA to generate A-Lipa KO mice. (**B**) Characterization of LIPA depletion at the mRNA (*n* = 7 in iWAT; *n* = 3 in BAT, eWAT and liver) and (**C**) protein levels in tissues from control (Ctrl) versus A-Lipa KO mice. (**D**) *Lipa* gene expression in homogenized eWAT separated by centrifugation into floating adipocyte and pelleted SV fractions from control (*n* = 6) and A-Lipa KO (*n* = 7) mice. (**E**) Schematic illustration of lipolysis assay by using genetic knockout mice model, A-Lipa KO mice, or pharmacological inhibition via lalistat-2 treatment. (**F**) Plasma FFA levels were measured at indicated time points in fasted A-Lipa KO and control mice (*n* = 12) (left) or in C57BL/6J mice fasted for 16 hours and injected intraperitoneally with 30 mg/kg lalistat-2 (*n* = 11) or vehicle (*n* = 10), followed by an additional 4 hours fasting. (**G**) Plasma FFA measured in A-Lipa KO and control mice (*n* = 4 each) fasted and individually housed at 4°C for indicated time points (left panel) or in C57BL/6J mice (*n* = 9 or 11) injected with 30 mg/kg lalistat-2 or vehicle 1 hour prior to fasting and individual housing at 4°C (right panel). (**H**) Plasma FFA (*n* = 4) measured at indicated time points in A-Lipa KO and control mice fasted for 16 hours then intraperitoneally injected with 1 mg/kg CL (left panel) or in C57BL/6J mice fasted for 16 hours, injected with 30 mg/kg lalistat-2 (*n* = 9) or vehicle (*n* = 8), and fasted for an additional 90 minutes prior to administration of 1 mg/kg CL without refeeding for indicated durations (right panel). All mice were male and fed an ND. Values are presented as mean ± SEM. Significant differences were determined by Student’s *t* test compared with their control group. *P < 0.05; **P < 0.01; ***P < 0.001. See also Supplemental Figures 4–10.

To directly test the functional role of adipose lysosomal lipolysis, we decided to use both pharmacologic and genetic approaches to LIPA inhibition. Lalistat-2 is a commonly used LIPA antagonist (26, 27) albeit with possible inhibitory overlap with other lipases (28). Thus, we additionally developed adipocyte-specific LIPA knockout (A-Lipa KO) mice using adiponectin-Cre–mediated excision of floxed LIPA alleles (Figure 2A). This mouse model developed significant depletion of LIPA transcript and protein levels specific to adipose tissue depots (Figure 2, B and C). In addition to adipocytes within adipose tissue, several other cell types in the SV fraction, such as macrophages, express LIPA and likely explain residual levels of tissue expression in the flox model. Dissolution of WAT to separate cells into floating (adipocyte) and pelleted (SV cell) fractions demonstrated a depletion specific to adipocytes (Figure 2D and Supplemental Figure 5). To characterize the overt effects of adipocyte-specific deficiency in lysosomal lipolysis, young mice fed a chow diet were examined, and both males (Supplemental Figure 6A) and females (Supplemental Figure 7A) had body weights similar to that of controls. Significant changes were neither observed at baseline in serum FFA, TG, or glucose levels (Supplemental Figure 6B and Supplemental Figure 7B) nor in food and water intake, oxygen consumption, respiratory quotient, and locomotor activity (Supplemental Figure 6, C–F, and Supplemental Figure 7C).

### Adipocyte LIPA deficiency disrupts lipolytic responses to fasting and CE.

The observed increases in LIPA expression under fasting and CE (Figure 1) led us to hypothesize that the role of LIPA in adipocyte lipolysis might manifest more overtly under such conditions. Both genetic (A-Lipa KO) and pharmacological (Lalistat-2) disruption of LIPA were compared in parallel to evaluate the function of lysosomal lipolysis (Figure 2E). A-Lipa KO mice had reduced plasma FFAs and glycerol following fasting (Figure 2F and Supplemental Figure 8A), which was similar to lalistat-2–treated mice in both males and females (Figure 2F, Supplemental Figure 8A, and Supplemental Figure 9A). Moreover, genetic and pharmacological disruption of LIPA resulted in blunted lipolysis upon acute CE (Figure 2G and Supplemental Figure 8B). Similar results were observed with CL administration, where A-Lipa KO and lalistat-2–treated mice had fewer plasma FFAs and glycerol compared with their controls (Figure 2H, Supplemental Figure 8C, and Supplemental Figure 9B). Since off-target effects for lalistat-2 on cytosolic lipases have been reported (28), we investigated its specificity in suppressing lysosomal lipolysis by treating explants of A-Lipa KO WAT with lalistat-2. An additional approximately 15% suppression of lipolysis was observed in the lalistat-2–treated group compared with the vehicle group (Supplemental Figure 10, A and B), suggesting that lalistat-2 primarily inhibits lysosomal lipolysis with lesser effects on cytosolic lipases. Thus, both our genetic and pharmacological approaches emphasize the importance of LIPA in fasting- and cold-induced lipolysis of adipocytes.

**Figure 3:**
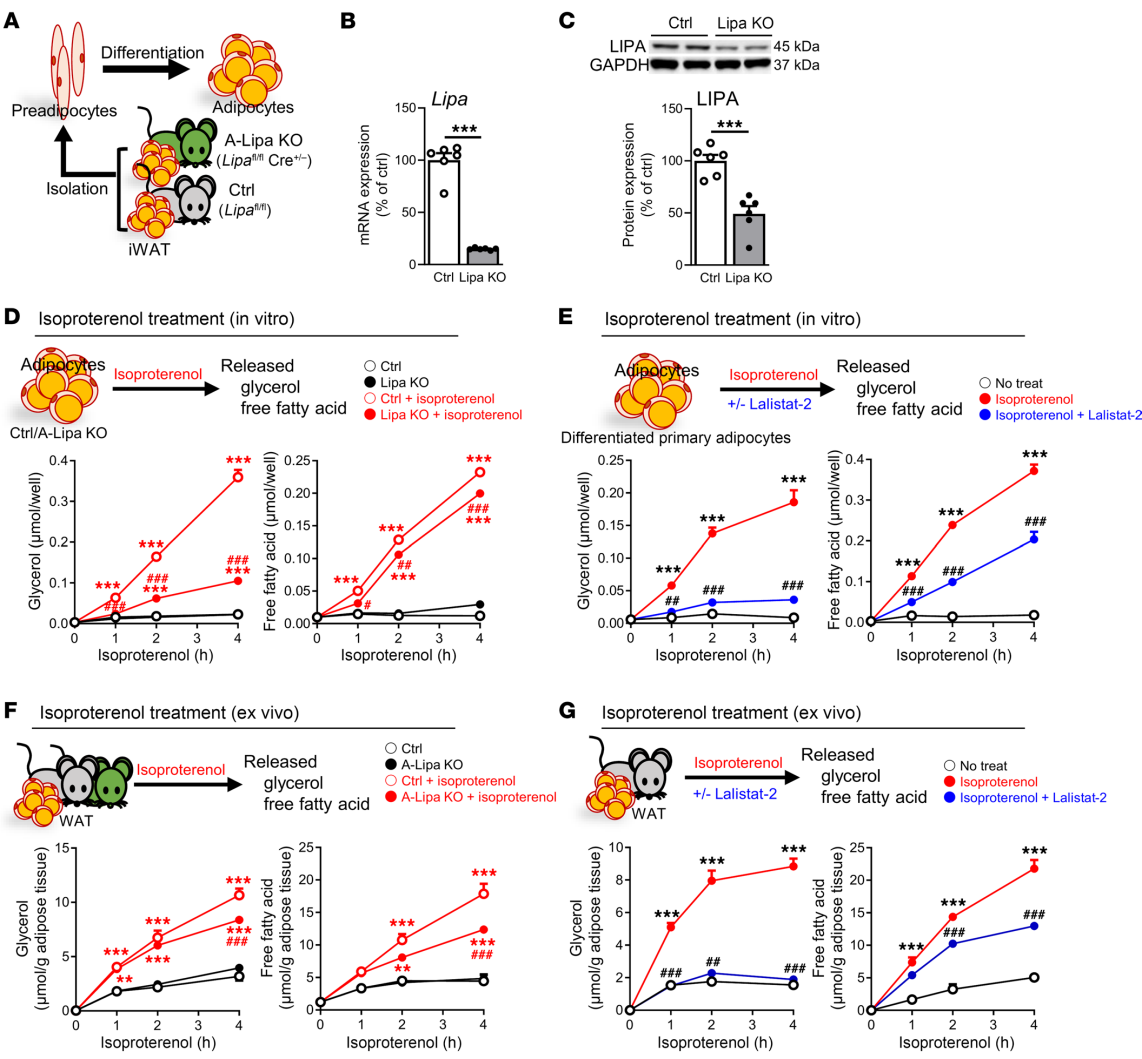
LIPA disruption suppresses β-agonist-induced adipose lipolysis in cultured adipocytes and WAT explant. (**A**) Schematic illustration of strategy to generate LIPA knockout primary adipocytes through differentiating the SV cells isolated from the iWAT of A-Lipa KO and control mice. (**B**) Confirmation of LIPA depletion at mRNA and (**C**) protein expression levels in Lipa KO adipocytes (*n* = 6). (**D**) Isoproterenol-induced lipolysis measured as glycerol and FFA release in supernatants from Lipa KO adipocytes (*n* = 6) or (**E**) lalistat-2–treated primary adipocytes (*n* = 3). (**F**) Glycerol and FFA levels of iWAT explant supernatant from A-Lipa KO versus control mice or (**G**) from lalistat-2– or vehicle-injected C57BL/6J mice treated with or without 1 μM isoproterenol at indicated time points (*n* = 4). All mice were male and fed an ND. Values are presented as mean ± SEM. Significant differences were determined by Student’s *t* test (**B** and **C**) or 2-way ANOVA with a post-hoc Tukey’s HSD test (**D**–**G**) for comparisons with the indicated groups (baseline groups: **P < 0.01; ***P < 0.001, or iso-treated groups: ^#^*P* < 0.05; ^##^*P* < 0.01; ^###^*P* < 0.001). See also Supplemental Figure 11–15.

Given the possibility for confounding effects of crosstalk between adipose tissue and other organs in vivo, we specifically dissected the role of LIPA and lipolysis in adipocytes using explants from murine WAT, cultured primary adipocytes, and C3H10T1/2 adipocytes. Nutrient-depleted eWAT explants from either A-Lipa KO mice or C57BL/6J mice treated with lalistat-2 released less glycerol and FFAs (Supplemental Figure 9C and Supplemental Figure 11A), results that were recapitulated in primary adipocytes and C3H10T1/2 adipocytes treated with lalistat-2 (Supplemental Figure 11, B and C). LIPA-deficient (Lipa KO) adipocytes were generated by isolating iWAT-derived SV cells from control and A-Lipa KO mice (Figure 3A) and significant reductions in LIPA protein and gene expression were confirmed (Figure 3, B and C). Lipolysis stimulated by isoproterenol or nutrient depletion was indeed lower in Lipa KO adipocytes (Figure 3D and Supplemental Figure 11D). To exclude potential effects of LIPA on adipocyte differentiation (19), we also generated tamoxifen-inducible LIPA-deficient primary adipocytes in culture by isolating iWAT-derived SV cells from mice harboring adiponectin-iCre/ERT2 and floxed LIPA alleles (Supplemental Figure 12A). No significant differences were observed in TG accumulation, 36B4 (*Rplp0*) gene expression, lactate dehydrogenase (LDH) release, or MTT assay between tamoxifen-treated primary adipocytes without adiponectin-iCre/ERT2 and the vehicle control (Supplemental Figure 13, A–D), suggesting that adipocyte function and survival were not affected by tamoxifen. Adipocytes with tamoxifen-induced LIPA knockout (TA-Lipa KO) exhibited a significant reduction in both LIPA protein and mRNA expression (Supplemental Figure 12, B and C), along with reductions in FFA and glycerol release during nutrient depletion or the presence of isoproterenol (Supplemental Figure 12, D and E). Both primary adipocytes and C3H10T1/2 adipocytes treated with lalistat-2 showed similar findings (Figure 3E and Supplemental Figure 14). Additionally, cultured iWAT explants from A-Lipa KO mice were unable to achieve isoproterenol-induced lipolysis at levels comparable to controls, an observation that was recapitulated in control explants treated with lalistat-2 (Figure 3, F and G). Similar to the results obtained from cultured WAT explants (Supplemental Figure 10), lalistat-2 further suppressed lipolysis by approximately 20% to 30% in Lipa KO adipocytes (Supplemental Figure 15), indicating that its lipolytic effects are primarily due to LIPA inhibition rather than cytosolic lipolysis. Overall, these observations highlight the importance of lysosomal lipolysis in liberating FFAs from adipocytes during fasting, CE, and β-receptor agonism.

### Adipocyte LIPA deficiency disrupts thermogenesis and oxygen consumption.

**Figure 4:**
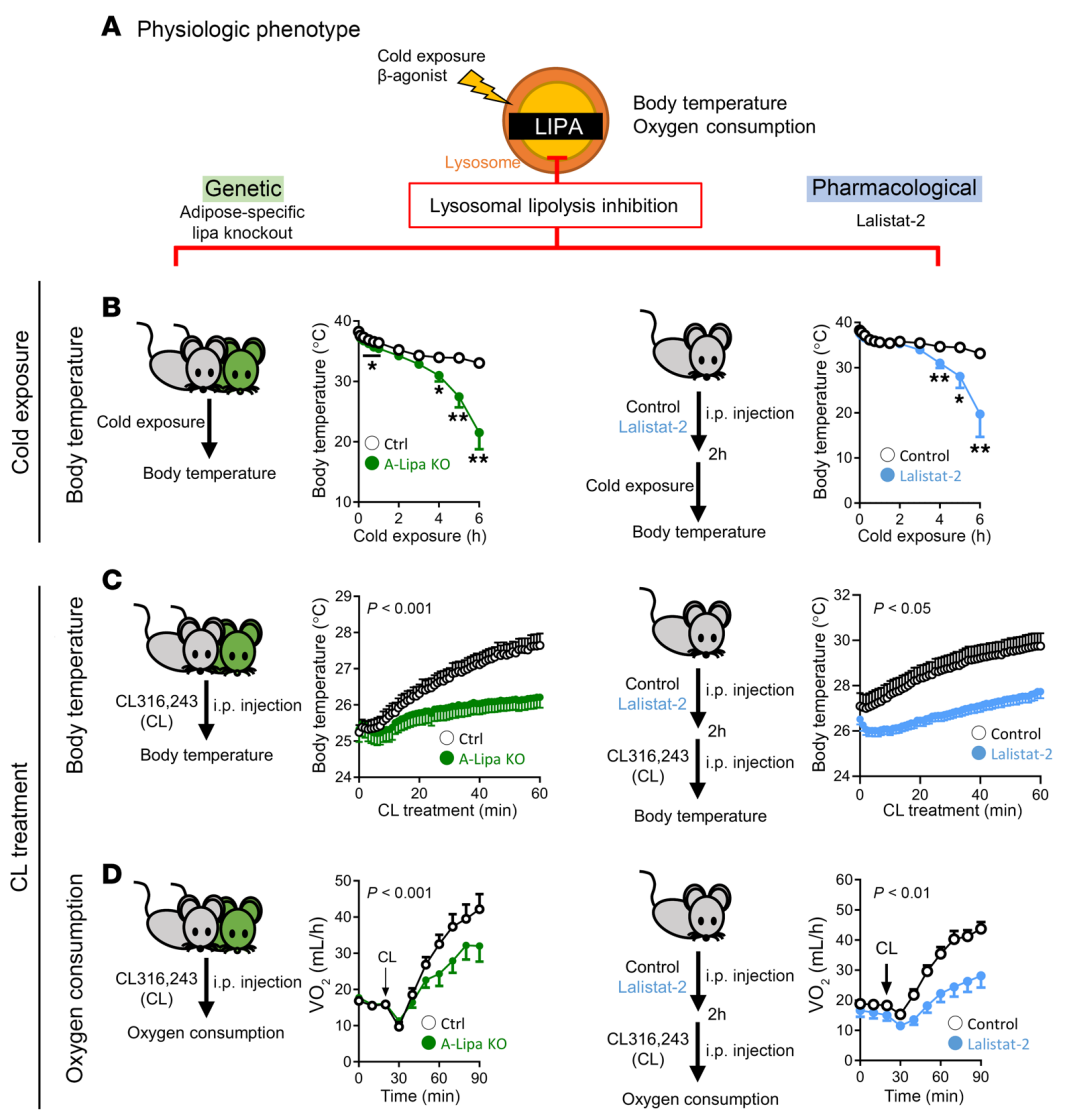
Adipocyte LIPA deficiency disrupts the regulation of body temperature and oxygen consumption upon CE and CL treatment. (**A**) Strategy to phenotype the role of LIPA in modulation of body temperature and oxygen consumption using genetic knockout or pharmacological inhibitor. (**B**) Core body temperatures monitored in A-Lipa KO and control mice (left panel) (*n* = 8 and 7) or in C57BL/6J mice (right panel) (*n* = 9 or 11) injected with 30 mg/kg lalistat-2 or vehicle 1 hour prior to indicated duration of individual housing at 4°C without food. (**C**) Body temperature (*n* = 10) and (**D**) oxygen consumption (*n* = 6) measured at indicated time points in A-Lipa KO and control mice intraperitoneally injected with 1 mg/kg CL or in C57BL/6J mice injected with 30 mg/kg lalistat-2 or vehicle 2 hours prior to CL treatment. All mice were male and fed an ND. Values are presented as mean ± SEM. Significant differences were determined by Student’s *t* test (**B**) or 2-way ANOVA (**C** and **D**) compared with baseline groups: *P < 0.05; **P < 0.01. See also Supplemental Figure 16.

As lipolysis-generated FFAs are a critical fuel source in settings of CE- and β-receptor–induced thermogenesis, we sought to use genetic and pharmacological inhibition to test the physiological relevance of LIPA in these scenarios (Figure 4A). At baseline, A-Lipa KO mice showed no significant differences in oxygen consumption (Supplemental Figure 6D) or body temperature (Supplemental Figure 6G). However, upon CE, both A-Lipa KO mice and lalistat-2–treated mice were unable to maintain core temperature during a cold tolerance test at 4ºC (Figure 4B and Supplemental Figure 16A). On the other hand, induction of thermogenesis initiated by CL316-243 demonstrated the inability of A-Lipa KO or lalistat-2–treated mice to respond with sufficient increases in body temperature increase (Figure 4C and Supplemental Figure 16B) and whole-body oxygen consumption (Figure 4D, Supplemental Figure 9D, and Supplemental Figure 16C). These data provide further evidence that adipocyte LIPA-mediated lipolysis contributes significantly to lipid-dependent physiological responses such a thermogenesis.

### Adipocyte LIPA expression is increased during obesity.

**Figure 5:**
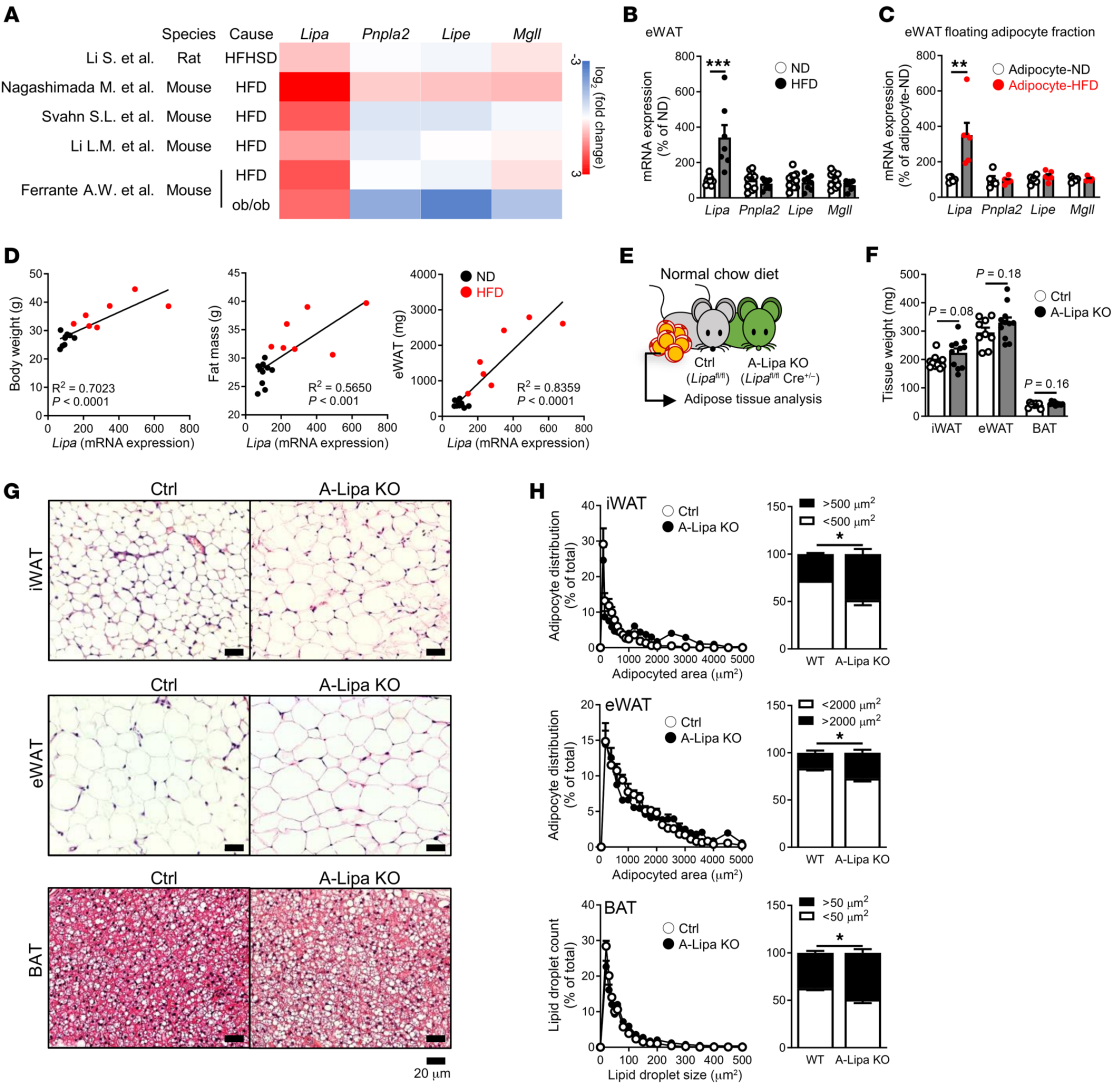
Adipocyte LIPA is associated with obesity and affects adipose tissue morphology. (**A**) Study information and log_2_ fold changes of lipase gene expression in indicated study datasets (22, 69–72) profiling eWAT from models of obesity including mice or rats fed high-fat high sugar diet (HFHSD) or HFD or those which were genetically predisposed (ob/ob model). (**B**) Gene expression of lipases in eWAT from ND- or HFD-fed mice (*n* = 10 or 8). (**C**) *Lipa* gene expression in adipocyte fractions isolated from eWAT of ND- or HFD-fed mice (*n* = 5 or 6). (**D**) Correlations between *Lipa* expression in eWAT and body weight (left panel), fat mass (middle panel), or eWAT weight (right panel) among ND and HFD-fed mice. (**E**) Experimental strategy outlining characterization of adipose tissue in A-Lipa KO and control mice. (**F**) Adipose tissue weight (*n* = 10 and 11) and (**G**) H&E-stained tissue sections of iWAT (top panel), eWAT (middle panel), and BAT (bottom panel) with (**H**) quantification of adipocyte size (*n* = 6) from A-Lipa KO and control mice at 16 weeks of age. Scale bars: 20 μm. All mice were male. Values are presented as mean ± SEM. Significant differences were determined by Student’s *t* test compared with control or ND groups: *P < 0.05; **P < 0.01; ***P < 0.001. The correlation (*r* square) and *P* value were calculated by Pearson’s *r* test. See also Supplemental Figures 17–19.

Obesity is associated with an increase in basal lipolysis (29), while there is some suggestion that cytosolic lipase expression decreases with obesity in humans (30) and mice (31, 32). Given the apparent roles of adipocyte lysosomal lipolysis in generating FFAs in healthy physiological responses, we hypothesized it might influence lipid storage with implications for the development of obesity and downstream pathologies. Examination of a broad set of publicly available datasets (Figure 5A) revealed *Lipa* mRNA was consistently upregulated in murine models of diet-induced obesity, whereas any changes in cytosolic lipase transcripts were milder and less consistent across studies. To further confirm these results in our diet-induced obesity mouse model, we fed C57BL/6J mice with a 60% HFD or normal chow diet (ND) for 12 weeks. *Lipa* expression in eWAT was increased with HFD, whereas expression of ATGL (*Pnpla2*), HSL (*Lipe*), or MGL (*Mgll*) was unchanged (Figure 5B).

In the context of obesity, Xu et al. has reported upregulation of a lysosomal biogenesis and lipid metabolism program within adipose tissue macrophages, including LIPA expression (22). Whether corresponding changes occur in adipocytes is unknown. We separated eWAT from ND- and HFD-fed C57BL/6J mice into floating adipocyte and pelleted SV fractions (Supplemental Figure 17). As shown in Figure 5C, *Lipa* gene expression, but not that of cytosolic lipases, was significantly upregulated in adipocytes from HFD-fed mice. Although macrophage markers were elevated only in the SV fraction (Supplemental Figure 17), we ruled out the possibility of macrophage contamination in the adipocyte fraction by injecting HFD-fed mice with clodronate (Supplemental Figure 18A) and showing upregulation of LIPA gene and protein expression remained unaffected (Supplemental Figure 18, B and C), also recapitulating our findings in Figure 5, B and C. These results suggest that macrophages play a minor role in adipose LIPA expression during obesity. Further, among mice fed ND or HFD, the gene expression of *Lipa* showed an excellent correlation with body weight, fat mass, and eWAT mass (Figure 5D), while no correlation was observed with expression levels of the cytosolic lipases (Supplemental Figure 19).

The positive correlation of LIPA expression with obesity led us to first evaluate the baseline features of adipose tissue from ND-fed A-Lipa KO mice (Figure 5E). No overt differences were noticed in the body weight (Supplemental Figure 6A) or the weights of adipose tissue including iWAT, eWAT, and BAT (Figure 5F) and nonadipose tissue such as liver and muscle (Supplemental Figure 6H). Interestingly however, histological analysis of A-Lipa KO mice showed larger adipocyte size in iWAT and eWAT tissue as well as a higher number of lipid droplets in BAT (Figure 5G with quantification of lipid droplet size displayed in Figure 5H).

### Adipocyte LIPA deficiency mediates the development of obesity and associated glucose intolerance.

**Figure 6:**
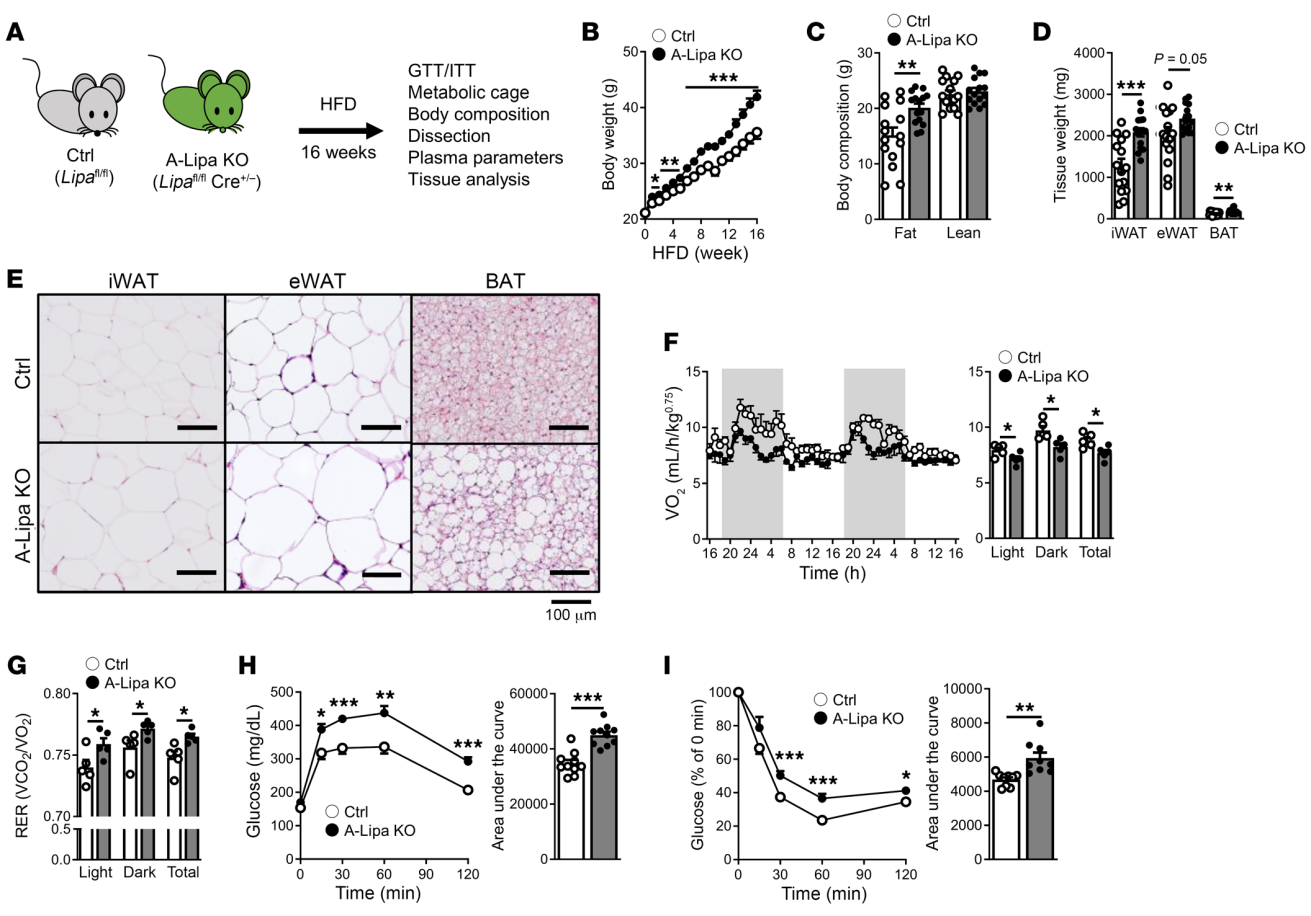
Adipocyte LIPA deficiency promotes the development of diet-induced obesity. (**A**) Experimental strategy outlining characterization of A-Lipa KO and control mice in response to diet-induced obesity with metabolic assays performed. (**B**) Body weight, (**C**) body composition, (**D**) adipose depot tissue weights, and (**E**) H&E-stained tissue sections of iWAT (left panel), eWAT (middle panel), and BAT (right panel) from A-Lipa KO and control mice after 16 weeks of HFD treatment (*n* = 15). Scale bars: 100 μm. (**F**) Oxygen consumption and (**G**) RER in A-Lipa KO and control mice 10 weeks into the HFD study (*n* = 5). (**H**) Glucose tolerance test and calculated area under the curve in A-Lipa KO and control mice assessed following 12 weeks of HFD (*n* = 10). (**I**) Insulin tolerance test and calculated area under the curve in A-Lipa KO and control mice assessed following 10 weeks of HFD (*n* = 9). All mice were male. Values are presented as mean ± SEM. Significant differences were determined by Student’s *t* test compared with ND groups: *P < 0.05; **P < 0.01; ***P < 0.001. See also Supplemental Figure 20.

To address the possible functional role of adipocyte LIPA in modulating obesity, mice with A-Lipa deficiency and littermate controls were fed a HFD for 16 weeks, and key metabolic outcomes were analyzed (Figure 6A). Mice lacking adipocyte LIPA gained significantly more weight on HFD (Figure 6B) which was primarily due to a higher fat mass without effect on whole-body lean mass (Figure 6, C and D) or muscle or liver mass, although hepatic TG content trended up but did not reach significance (Supplemental Figure 20, A and B). At the level of adipose tissue, iWAT and BAT from HFD-fed A-Lipa KO mice were heavier and larger and there was a trend for increase in eWAT that did not reach significance (Figure 6D). In addition, all adipose depots, iWAT, eWAT, and BAT, developed adipocyte hypertrophy (Figure 6E), indicating a direct role for adipocyte lysosomal lipolysis in regulating lipid storage. To test the physiological mechanisms responsible for increased adiposity in mice, we performed metabolic cage studies and determined A-Lipa KO mice had blunted oxygen consumption (Figure 6F) and elevated respiratory exchange ratio (RER) (Figure 6G and Supplemental Figure 20C), consistent with the concept of impaired lipid oxidation, and these changes could not be explained by the lack of differences in locomotor activity (Supplemental Figure 20D) and food intake (Supplemental Figure 20E). We also performed glucose and insulin tolerance tests to determine whether the lack of adipocyte lysosomal lipolysis and associated defects would ultimately manifest in diabetic pathology. A-Lipa KO mice fed a HFD had worse glucose and insulin tolerance (Figure 6, H and I) along with lower plasma adiponectin levels (Supplemental Figure 20F) compared with control mice. The summation of these phenotypes highlights an essential role for adipocyte LIPA in mediating obesity-related TG storage, lipolysis, and metabolic outcomes.

### Adipocyte LIPA deficiency has no suppressive effects on the cytosolic lipolysis machinery.

**Figure 7:**
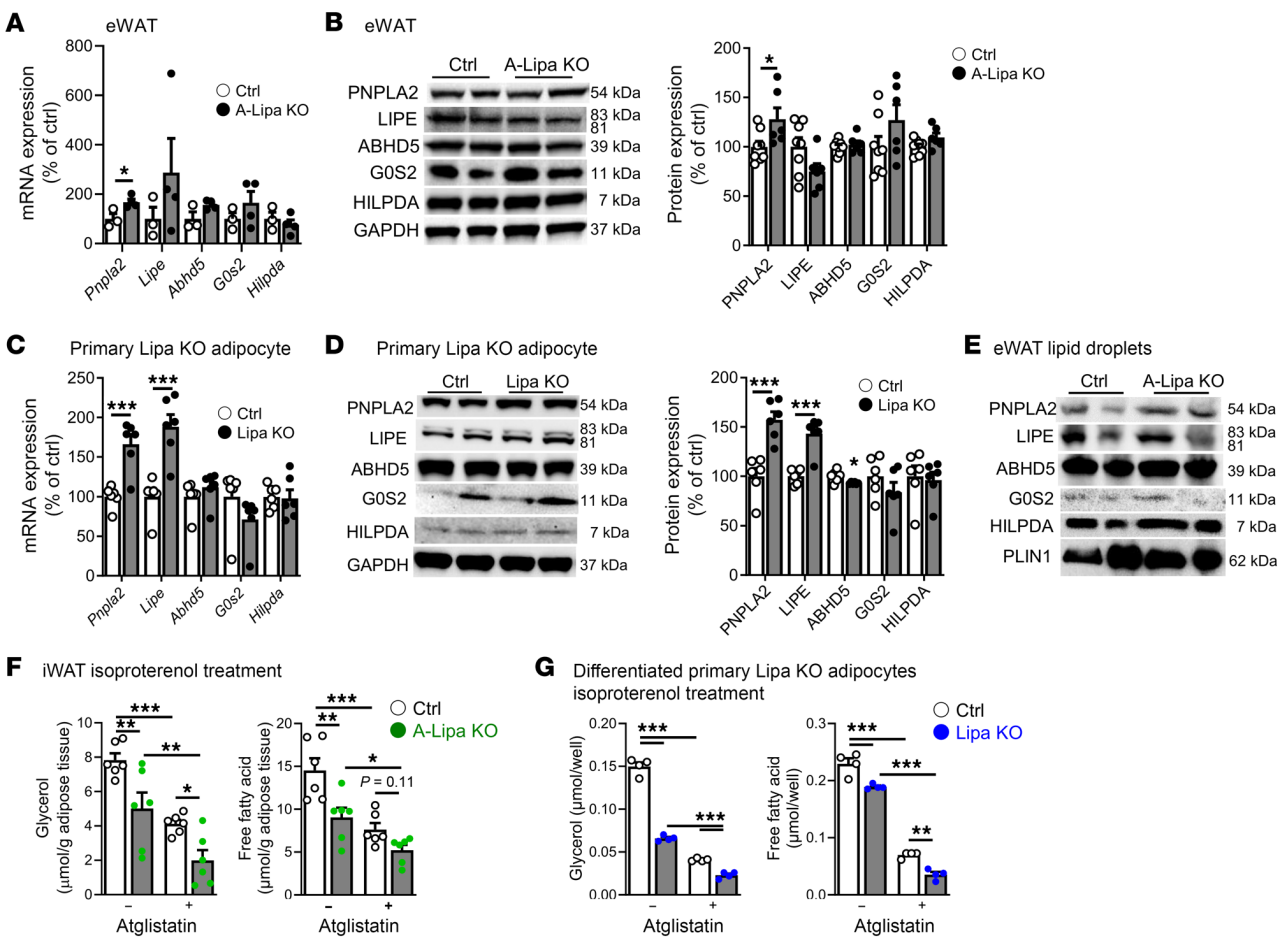
Lipolysis mediated by LIPA is independent of ATGL. (**A**) Expression of mRNA (*n* = 3–4) and (**B**) protein (*n* = 6–8) in lipases, ATGL (PNPLA2), and HSL (LIPE), and ATGL-related cofactors G0S2, HILPDA, and CGI-58 (ABHD5) in eWAT from A-Lipa KO and control mice at 12 weeks old. (**C**) Expression of mRNA and (**D**) protein in lipases, ATGL (PNPLA2), and HSL (LIPE), and ATGL-related cofactors G0S2, HILPDA, and CGI-58 (ABHD5) in Lipa KO adipocytes (*n* = 6). (**E**) Protein expression of lipases, ATGL (PNPLA2), and HSL (LIPE), and ATGL-related cofactors G0S2, HILPDA, and CGI-58 (ABHD5) in lipid droplets isolated from eWAT of A-Lipa and control mice at 12 weeks old. (**F**) Glycerol and FFA release into the supernatant of isoproterenol-stimulated iWAT explants from A-Lipa KO versus control mice with or without atglistatin cotreatment (*n* = 6). (**G**) Glycerol and FFA release into the supernatant of isoproterenol-stimulated Lipa KO versus control adipocytes with or without atglistatin cotreatment (*n* = 4). All mice were male. Values are presented as mean ± SEM. Significant differences were determined by Student’s *t* test (**A**–**D**) or by 2-way ANOVA with a post hoc pairwise *t* test (**F** and **G**) for comparisons with the indicated groups: *P < 0.05; **P < 0.01; ***P < 0.001. See also Supplemental Figures 21 and 22.

With prior reports having implicated ATGL/PNPLA2 in upstream regulation of lipophagy in hepatocytes (16, 33), we desired to determine the interdependence of lysosomal and cytosolic lipolysis. We first assessed the expression of cytosolic lipases, ATGL and HSL, and ATGL cofactors such as the suppressors G0S2 and hypoxia-inducible lipid droplet–associated (HILPDA), or the activator CGI-58 (ABHD5) (3), in A-Lipa KO mice. No reductions in the transcript levels and protein expression of ATGL (PNPLA2), HSL (LIPE), or the ATGL regulators were noted in the absence of LIPA (Figure 7, A and B). Instead, we noted slight increases in both mRNA and protein expression of ATGL (PNPLA2), perhaps as a compensatory response (Figure 7, A and B). In order to ascertain whether any expression changes were specific to adipocytes, we separated the eWAT into adipocytes and SV cells. The expressions of ATGL (*Pnpla2*), HSL (*Lipe*), CGI-58 (*Abhd5*), and *G0s2* were increased in A-Lipa KO adipocyte fractions, whereas *Hilpda* transcripts were unchanged (Supplemental Figure 21A). Our assessment of the same cytosolic lipolytic markers in iWAT from A-Lipa KO mice showed similar results to eWAT with selectively increased ATGL (PNPLA2) expression (Supplemental Figure 21, B and C). We also used the Lipa KO adipocytes described in Figure 3A and noticed increased ATGL (PNPLA2) and HSL (LIPE) in both gene and protein expression with a slight but significant reduction of CGI-58 (ABHD5) protein expression (Figure 7, C and D). Finally, using the same tamoxifen-inducible LIPA-deficient primary adipocytes described above (TA-Lipa KO), we observed slight but significant increases in ATGL (*Pnpla2*), HSL (*Lipe*), and CGI-58 (*Abhd5*) transcripts coincident with rises in the respective proteins (Supplemental Figure 12, F and G). These findings demonstrate that in the setting of LIPA deficiency, expression of the cytosolic lipolytic machinery in adipocytes is clearly not suppressed and even sees variable levels of elevation, possibly as a compensatory response to the loss of lysosomal lipolysis.

To further determine whether the absence of LIPA influences the recruitment of cytosolic lipases to lipid droplets, we isolated lipid droplets from the eWAT of A-Lipa KO and control mice and assessed the presence of ATGL, HSL, and related cofactors. Only ATGL (PNPLA2) was slightly increased on eWAT lipid droplets, whereas levels of HSL (LIPE) and the ATGL cofactors CGI-58 (ABHD5), G0S2, and HILPDA were unchanged (Figure 7E). Coupled with the gene and protein expression results above, these data support the notion that the phenotypes observed when LIPA and lysosomal lipolysis are perturbed are not confounded by declines in the expression or localization of the cytosolic lipolysis machinery; rather, signatures of a mild compensatory elevation in ATGL and its cofactors are observed. Finally, to examine the interdependence of LIPA and ATGL on adipocyte lipolysis, atglistatin, an ATGL-specific antagonist able to suppress lipolysis on par with what occurs in adipose-specific ATGL knockout mice (34), was used to evaluate the combined effect of ATGL inhibition in LIPA-deficient WAT explants and adipocytes. Lipolysis suppressed in LIPA-deficient WAT explants was enhanced in the presence of atglistatin, surpassing the effect of atglistatin alone upon stimulation with isoproterenol or nutrient depletion (Figure 7F and Supplemental Figure 22A). Similar results were recapitulated in Lipa KO adipocytes (Figure 7G and Supplemental Figure 22B), suggesting independent and additive effects between lysosomal/LIPA-regulated and cytosolic lipolysis.

## Discussion

Our findings reveal a critical role for the adipocyte lysosomal system in mediating TG lipolysis with broad relevance to fasting, thermogenesis, and diet-induced obesity. Prior studies of global LIPA deletion have noted adipose phenotypes such as depletion of WAT and BAT (19) and defective thermogenesis (18). However, the direct interpretation of these studies was difficult in light of known roles for LIPA in enteric (35), hepatic (36), and endothelial (37) systems to which adipose phenotypes could be secondary. Observation of defects in TG lipolysis with specific deletion of adipocyte LIPA in our study solidifies the importance of adipocyte lysosomal lipolysis in this setting and clarifies the net directional impact of its role as leading to consistently increased adipocyte TG accumulation.

The relationship between lysosomal lipolysis mediated by LIPA and cytosolic lipolysis mediated by ATGL, HSL, and MGL has been shown to be complex in other studies (38), and which enzymes truly and directly mediate TG hydrolysis, especially in complex systems, is difficult to evaluate. Although our findings provide evidence that LIPA has important and independent contributions to overall adipocyte lipolysis above and beyond those mediated by ATGL and classical cytosolic lipolysis, these 2 lipolytic systems also appear to cooperate in ways that are difficult to deconvolve. For example, deletion of autophagy components may interfere with observed scaffolding roles for LC3 in recruiting ATGL to the lipid droplet (17). Additionally, FFAs derived from lysosomal lipolysis are known to activate peroxisome-proliferator–activated receptor signaling in other cell types with numerous downstream effects on cellular metabolism (39). As such, phenotypes observed with LIPA deficiency in adipocytes could be partially explainable by an indirect regulation of cytosolic lipolysis. On the other hand, a growing body of work increasingly suggests the converse phenomenon: that modulation of cytosolic lipase activity has second-order effects on the autophagy-lysosome system that are required for the ultimate effects on lipid metabolism. For example, Sathranarayan et al. studied hepatocytes and demonstrated inhibition of ATGL-reduced lipolysis in a manner dependent on second-order regulation of lipophagy (16, 33). Similarly, it was recently demonstrated that ATGL acts in hepatocytes to reduce lipid droplets to a small size palatable to lipophagy, upon which full lipid droplet catabolism ultimately depends (16). In adipocytes, micro-lipid droplets are known to form during prolonged lipolysis (40) and may be similarly targeted for lipophagy. This fits our observations that increased adipocyte LIPA expression is especially a feature of CE, fasting, and β-receptor agonist induced lipolysis, whereas we observed less consistent expression changes in cytosolic lipases that are known to have posttranslational regulation and display acute responses (41). Last, 2 studies have demonstrated that deficiency of ATGL (9) or its activator, ABHD5/CGI-58 (42), in brown adipocytes had no effect on thermogenesis, suggesting the crucial role of alternative mechanisms of brown adipocyte lipolysis such as lipophagy. Interpreted in any relation to cytosolic lipolysis, our results highlight dramatic roles for LIPA in mediating adipocyte lipolysis, and these interactions continue to deserve investigation.

The mechanism of delivery of lipids to the lysosome for LIPA-mediated hydrolysis is an ongoing area of interest. Although a recent report showed that lipid droplets could directly interact with lysosomes (43), autophagy nevertheless is a well-examined and currently the major mechanism for delivering lipid droplets into lysosomes (43–45). For example, Singh et al. have shown autophagy is required for lipid droplet breakdown in hepatocytes (14). Adipose-specific ATG7 knockout mice had lower plasma FFA and glycerol levels on the nonfasting baseline and isoproterenol-stimulated condition (46). In addition, chaperone-mediated autophagy has been shown to participate in lipolysis by targeting perilipin 2 and 3, a family of lipid droplet–associated proteins, to precede lipolysis (47). However, autophagy also mediates degradation of numerous other cellular cargo, such as dysfunctional mitochondria, which may also contribute to these phenotypes. Indeed, one such study demonstrated adipocyte-specific deletion of 2 other autophagy genes, ATG3 and ATG16L, both resulting in accumulation of dysfunctional mitochondria (48). This was shown to drive increases in lipid peroxides loaded in low-density lipoprotein, resulting in hepatic insulin resistance along with mild defects in thermogenesis. At present, it remains difficult to determine the relative contributions of defective lipolysis versus accumulation of dysfunctional mitochondria to the complex metabolic phenotypes observed with adipocyte-specific autophagy deficiency. Although we have demonstrated the fundamental role of adipocyte lysosomal lipolysis in lipid homeostasis, basic mechanisms mediating truly selective targeting of lipid droplets for lipophagic degradation remain poorly defined (38) and developments in this area will enable further study of the physiological roles of lipophagy in adipocytes.

Beyond its roles in TG hydrolysis, LIPA is also crucial for cholesterol ester hydrolysis in the lysosome (19, 49). One recent study observed that adipocyte-specific LIPA overexpression in mice could reduce adipose lipid accumulation, but induced only modest and nonsignificant increases in TG lipolysis in the studied model (50). While we demonstrated the essentiality of LIPA for full adipocyte TG lipolysis, the lack of major increase with overexpression in that study may be due to a requirement for upstream increases in TG delivery, as well as more than a mild increase in LIPA expression achieved with the transgenic model to fully harness the potential. This study also posited the impact of free cholesterol generation and consequent sterol regulatory element-binding protein regulation and steroidogenesis as important roles for LIPA in regulating adipocyte phenotype. While these changes could plausibly explain some metabolic phenotypes, we observed acute effects of lalistat-2 on TG lipolysis that are unlikely to be dependent on transcriptional regulation as well as cell-intrinsic reductions in TG lipolysis, which were highly consistent with lalistat-2 inhibition or LIPA deficiency in several models. Future studies might delve more in depth on the impact of lysosome-generated FFA on transcriptional regulation of mitochondrial and metabolic genes, along with the potential for LIPA-generated FFA to participate in TG-FFA cycling (51) or direct UCP1 activity regulation (52) relevant to thermogenesis. Overall, by using several models of pharmacological and genetic ablation of LIPA in adipocytes, we have demonstrated crucial roles for this enzyme in mediating adipocyte lipolysis and that it impacts thermogenesis, fasting, and the development of obesity, sparking further study of how this process occurs and might be harnessed to better understand disease.

## Methods

### Sex as a biological variable.

Our study predominantly focused on male mice, as the hormonal fluctuations associated with the estrous cycle in females could influence systemic lipid metabolism (53, 54). Nevertheless, we also performed key lipolysis experiments in female mice, which recapitulated the results observed in males. These findings suggest that the contributions of lysosomal lipolysis in adipose tissue are relevant to both sexes.

### Animals.

Five-week-old C57BL/6 male mice were purchased from the Jackson Laboratory. All mice were housed at 23 ± 1°C and maintained on a 12-hour light/12-hour dark cycle. In all of the experiments, mice were fed a commercial chow diet (Purina 5053) until 8 weeks of age, and then the mice were randomly divided into groups for experiments. *Lipa*-floxed mice were provided in house (36).

### Strategy and generation of A-Lipa and TA-Lipa KO mice.

*Adipoq*-Cre BAC and *Adipoq*-icre/ERT2 transgenic mice (RRID: IMSR_JAX:010803 and 025124) were purchased from Jackson Laboratory. *Adipoq*-Cre BAC and *Adipoq*-icre/ERT2 transgenic mice, in which classic or tamoxifen-inducible codon-improved Cre recombinase is specifically expressed in adipose tissue, were bred with homozygous-floxed Lipa mice (*Lipa*^fl/fl^) to generate *Lipa^fl/+ Cre+^* or *Lipa^fl/+ iCre/ERT2+^* progeny. These mice were then backcrossed to *Lipa*^fl/fl^ mice. *Lipa*^fl/fl Cre–^ (control) and *Lipa*^fl/fl Cre+^ (A-Lipa KO) or *Lipa*^fl/fl iCre/ERT2+^ mice (TA-Lipa) were further used for experiments or generating their progeny.

### Mouse phenotyping.

For examining the influence of adipose-specific LIPA on the metabolic state, mice were fed a chow diet until indicated age for experiments. In the HFD treatment experiment, mice were fed HFD containing 60% kcal fat (D12492, Research Diet) for 16 weeks. For glucose tolerance tests, mice were fasted for 6 hours and a blood sample was taken from the mouse tail vein (0 minute point). Subsequently, mice were intraperitoneally treated with glucose solution (1 g of glucose [G8270, Sigma-Aldrich]/ 10 mL of sterile PBS, 10 μL/g body weight) and blood samples were collected at different time points (15, 30, 60, and 120 minutes). For insulin tolerance tests, blood samples were collected from the mouse tail vein (0 minute point) and the mice were then intraperitoneally injected with insulin solution (0.1 U of insulin/mL of sterile PBS, 10 μL/g body weight) after fasting for 6 hours. The blood samples were taken at 15, 30, 60, and 120 minutes. The oxygen consumption was measured using an indirect calorimetric system (PhenoMaster, TSE systems). Mice were tested for 24 hours for O_2_ consumption, CO_2_ production, and activity assessed as beam breaks (Systems LabMaster software, version 4.8.7). The RER, oxygen consumption, and activity were determined using LabMaster software, version 4.8.7. Data were analyzed as the average over 12-hour dark or 12-hour light cycle intervals. Cumulative food intake was assessed in individually housed mice daily for 14 days total, taking into account spilled food. Body composition was determined by EchoMRI-100H (EchoMRI LLC) and presented as fat and lean mass, in accordance with the manufacturer’s instructions. For studies administering CL (149910, Tocris Bioscience), mice were fasted for 16 hours and then intraperitoneally injected with 1 mg/kg CL (0.1 g of CL/ 1 mL of sterile PBS, 10 μL/g body weight). For experiments assessing acute changes in oxygen consumption and body temperature upon CL administration, mice were anesthetized with ketamine cocktail (5 μL/kg body weight composed of 20 mg/mL ketamine hydrochloride [NDC 50989-161-06, Vedco] and 4 mg/mL xylazine [NDC 46066-750-02, Pivetal] in sterile PBS). For monitoring mouse body temperature, IPTT-300 transponders (Bio Medic Data Systems) were used following the manufacturer’s instructions. Briefly, mice were injected with a temperature transponder (IPTT-300) interscapularly in advance and mouse body temperature was measured by an IPTT-300 reader (DAS-8027IUS-A) under indicated conditions with ketamine anesthesia. Lalistat-2 (SML2053, Sigma-Aldrich) was dissolved in DMSO (300 mg/mL) and, after mixing with 1 volume of polyethylene glycol 300 (P0108, Spectrum Chemical), 8 volumes of sterile PBS were added and gradually vortexed. Mice received lalistat solution (30 mg/mL of PBS, 10 μL/g body weight) under indicated conditions. Mice were injected daily intraperitoneally with 0.6 mg clodronate (CLD-8909, Encapsula NanoSciences LLC, TN, USA) for 2 days followed by indicated conditions.

### Cell culture.

C3H10T1/2 clone 8 (ATCC CCL-226) cells were purchased from ATCC. Primary adipocytes were provided in house (55). TA-Lipa KO adipocytes were generated as previously described (55).

Two days after reaching confluence (D0), C3H10T1/2, primary adipocytes, Lipa KO, or TA-Lipa KO adipocytes were incubated in a differentiation medium containing 0.25 μM dexamethasone, 10 μg/mL insulin, 0.5 mM 3-isobutyl-1-methylxanthine, and 1 μM rosiglitazone in the growth medium. After 48 hours (D2), the cell culture medium was changed to postdifferentiation medium containing 5 μg/mL insulin in the growth medium, and a fresh postdifferentiation medium was also supplied every other day until experiments (D8–D10, unless otherwise noted). For inducing LIPA knockout, TA-Lipa KO adipocytes were incubated with10 μM tamoxifen containing 5 μg/mL insulin in the growth medium between D4 and D6, which was changed to postdifferentiation medium containing 5 μg/mL insulin until experiments (D8).

### Histological analysis and tissue weights.

BAT, iWAT, eWAT, liver, and gastrocnemius muscles were weighed upon mouse dissection. Tissue samples of BAT, iWAT, and eWAT were fixed in 4% paraformaldehyde overnight and then transferred to 70% ethanol. The fixed samples were embedded in paraffin for staining with H&E.

### Analysis of plasma metabolites and hormones.

Glucose, TG, and FFA were determined using commercially available kits: Autokit Glucose (NC9927772, Wako Chemicals), Infinity Triglyceride Reagent (TR22421, Thermo Fisher), NEFA HR(2) (Reagent 1 [434-91795] and Reagent 2 [436-91995], Wako Chemicals), and adiponectin/Acrp30 Quantikine ELISA kit (R&D Systems), respectively. All kits were used in accordance with the manufacturer’s instructions.

### Hepatic lipid analysis.

To quantify hepatic TG content, the lipids were extracted by 1.2 mL of hexane/2-propanol (3:2 v/v) for each 50 mg of liver samples. The liver samples were homogenized and centrifuged at 4°C for 10 minutes at 10,000*g*. After evaporating the solvent, the lipid extracts were resuspended in 10% Triton X-100 in 2-propanol with sonication and determined enzymatically using the Infinity Triglyceride Reagent (TR22421, Thermo Fisher).

### Quantification of gene expression.

RNA was isolated with QIAzol Lysis Reagent (79306, QIAGEN) and then reverse transcribed using SuperScript VILO (Thermo Fisher). For quantifying the mRNA expression level, real-time PCR was performed using a LightCycler system (QuantStudio) using SYBR Green fluorescence signals (SYBR Select Master Mix, 4472908, Thermo Fisher). The protocol for amplification was as follows: denaturation, 95°C for 1 minute; annealing, 60°C for 10 seconds; extension, 72°C for 40 seconds. All measured gene expression was normalized to the levels of ribosomal protein lateral stalk subunit P0 (*Rplp0*). Primer sequences are provided in Supplemental Table 1.

### Western blotting.

Cells or tissues were lysed in a standard RIPA lysis buffer. Standard techniques were used for protein quantification, separation, transfer, and blotting (56). The following primary antibodies were used: LIPA (1:1000, NBP1-54155, Novus Biologicals), ATGL/Pnpla2 (1:1000, 2138S, Cell Signaling Technology), HSL/Lipe (1:1000, 4107S, Cell Signaling Technology), CGI-58/ABHD5 (1:1000, 12201-1-AP, Proteintech), G0S2 (1:1000, 12091-1-AP, Proteintech), HILPDA (1:200, sc-376704, Santa Cruz Biotechnology Inc.), GAPDH (1:2000, ab-22555, Abcam), ITGAM (1:1000, 17800S, Cell Signaling Technology), ADGRE1 (1:1000, 70076S, Cell Signaling Technology), and PLIN1 (1:1000, 9349S, Cell Signaling Technology).

### Adipocyte sizing and lipid content analyses.

A minimum of eight 20× fields per mouse were imaged in iWAT, eWAT, and BAT H&E-stained slides by a blinded observer at identical settings. Adipocyte sizing analyses were performed using the Adiposoft plugin for ImageJ software (NIH), version 1.54.

### Ex vivo and in vitro lipolysis assays.

Methods for the ex vivo and in vitro lipolysis from adipose tissues or adipocytes in fasting- and isoproterenol-stimulated conditions were adapted from Enoksson et al. (57). Briefly, eWAT and iWAT were removed from anesthetized animals and immediately placed in Krebs-Henseleit buffer (KHB, pH 7.4 at 37°C) supplemented with 2% fatty acid-free BSA (AK8909, Akron Biotech), 2.5 mM glucose (25-037-CI, Corning), and 5 mM HEPES (H0887, Sigma-Aldrich). WATs from the same genotype mice were combined and minced into small pieces. Approximately 50 mg eWAT was placed into 500 μL/well nutrient-free medium (EBSS [E2888, Sigma-Aldrich] [1 g/L glucose] with 2% fatty acid-free BSA) and approximately 50 mg iWAT was placed into 500 μL/well KHB with supplements above with or without 1 μM isoproterenol, respectively. As with the ex vivo lipolysis assay, cells used for in vitro lipolysis were washed twice with sterile PBS, and after carefully removing all the residual liquid in wells, EBSS (1 g/L glucose) contained with 2% fatty acid–free BSA or the KHB with supplements above with/without 1 μM isoproterenol was added to appropriate wells. The tissues or cells for ex vivo or in vitro lipolysis assays were kept at 37°C in 5% CO_2_, and the supernatant was collected at the indicated time points without collecting the tissues or disrupting the cells. The glycerol and FFA levels used as the lipolysis markers were measured by Glycerol Assay Kit (MAK117, Sigma-Aldrich) and NEFA HR(2) (Reagent 1 [434-91795] and Reagent 2 [436-91995], Wako Chemicals) in accordance with the manufacturer’s instructions. All the results were normalized and presented as concentration per mg WAT.

### Isolation of lipid droplet protein.

Lipid droplet isolation from WAT was as described before with some modifications (57–59). Briefly, eWAT or iWAT were removed from anesthetized animals, rinsed in PBS, minced, and suspended in a homogenization buffer (250 mM sucrose, 20 m M Tris-HCl, 1 mM EDTA, pH 7.4 with protease inhibitor) followed by homogenization with Kinematica PT 2100 (14,000 rpm, 30 seconds) and centrifugation (1,000*g*, 10 minutes) to remove the nuclei. The supernatant was centrifuged further (14,000*g*, 20 minutes), the floating lipid droplet layer washed before resuspension in lysis buffer containing 2% SDS, and delipidated using trichloroacetic acid/acetone to isolate the lipid droplet proteins for subsequent Western blotting.

### RNA-seq dataset.

Transcriptomics data from previous studies were obtained from GEO and analyzed. Microarray-based data (Figure 1B: M. Defour et al., GSE154612 (60); M. Schupp et al., GSE46495 (61); M. Ibrahim et al., GSE118978 (62); Y. Nakai et al., GSE7623 (63); Figure 1E: X. Liu et al., GSE2674; J. Lee et al., GSE72210 (64); B. Shan et al., GSE169672 and GSE165974 (65); Y. Li et al., GSE119452 (66); P. Yu et al., GSE118849 (67); J. Rodo et al., GSE148361 (68); Figure 5A: S. Li et al., GSE8700 (69); M. Nagashimada et al., GSE167311 (70); S.L. Svahn et al., GSE79434 (71); L.M. Li et al., GSE77943 (72); A.W. Ferrante et al., GSE8831 (22) were extracted from GEO using the GEO2R online tool (73).

### Statistics.

All of the results are given as the mean ± SEM. Bars in graphs represent standard errors, and significance was assessed by Student’s 2-tailed *t* tests or 1- or 2-way ANOVA with a post hoc Tukey’s honestly significant difference (HSD) test or pairwise *t* test as denoted in each figure legend. Differences with *P* < 0.05 were considered statistically significant.

### Study approval.

Studies involving mice were performed under protocols approved by the Animal Studies Committees at the University of Pittsburgh School of Medicine and Washington University School of Medicine.

### Data availability.

RNA-Seq data are obtained from GEO as described in the RNA-Seq datasets. Values for all data points in graphs are reported in the Supporting Data Values file. Any additional information required to reanalyze the data reported in this paper is available upon request.

## Author contributions

YSY performed the majority of experiments. YSY and TDE wrote the initial manuscript. MI, SJJ, XZ, ZL, AP, AG, and BD assisted with cell culture and animal experiments and analyses. TDE, MI, AJ, DK, SK, TG, HZ, PD, FJS, ACS, JC, IJL, and BR provided reagents, expertise, feedback, and conducted critical editing of the manuscript. BR conceived and supervised the project.

## Supplementary Material

Supplemental data

Unedited blot and gel images

Supporting data values

## Version 1 03/17/2025

Electronic publication
