# Supplementary material for: Identification of lysosomal lipolysis as an essential noncanonical mediator of adipocyte fasting and cold-induced lipolysis
Source: J Clin Invest. 2025 Mar 17;135(6):e185340. doi: 10.1172/JCI185340 (PMC11910232; doi:10.1172/JCI185340)
Supplement: Supplemental data [file jci-135-185340-s192.pdf]

Identification of Lysosomal Lipolysis as a Non-canonical Mediator of Adipocyte Fasting and Cold-induced Lipolysis

Yu-Sheng Yeh[1,3], Trent D. Evans[4], Mari Iwase[5,14], Se-Jin Jeong[4], Xiangyu Zhang[1,3], Ziyang Liu[1,3], Arick Park[4], Ali Ghasemian[4], Borna Dianati[4], Ali Javaheri[4,9], Dagmar Kratky[10], Satoko Kawarasaki[11], Tsuyoshi Goto[11,12], Hanrui Zhang[13], Partha Dutta[1,3], Francisco J. Schopfer[2], Adam Straub[2], Jaehyung Cho[6,8], Irfan Lodhi[7], Babak Razani[1,3*]

I. Supplemental Table 1

II. Supplemental Figures 1 – 22

Table S1

Gene	Gene ID	Forward	Reverse
Rplp0	11837	ATCCCTGACGCACCGCCGTGA	TGCATCTGCTTGGAGCCCACGTT
Lipa	16889	CGTGGGCGGAAGAACCATT	AGCAAGCCGTGCTGAAGAT
Pnpla2	66853	TAATGTTGGCACCTGCTTCA	CCACTCACATCTACGGAGCC
Lipe	16890	GGAGAGAGTCTGCAGGAACG	CCTGCAAGAGTATGTCACGC
Mgll	23945	CACTTTTCCAGAACACACCC	TGACTTTGCTCGGGGACC
Abhd5	67469	TGTTTGAAGATGACACGGTGA	ACCTATCCGCTGAAGCATTG
G0s2	14373	AGTGCTGCCTCTCTTCCCAC	TCCTGCACACTTTCCATCTG
Hilpda	69573	TCGTGCAGGATCTAGCAGCAG	GCCCAGCACATAGAGGTTCA
Adipoq	11450	TACAACCAACAGAATCATTATGACGG	GAAAGCCAGTAAATGTAGAGTCGTTGA
Lep	16846	TTCACACACGCAGTCGGTATC	GGCTGGTGAGGACCTGTTG
Itgam	16409	CCATGACCTTCCAAGAGAATGC	ACCGGCTTGTGCTGTAGTC
Itgax	16411	TGGGGTTTGTTTCTTGTCTTG	GCCTGTGTGATCGCCACATTT
Adgre1	13733	TTTCCTCGCCTGCTTCTTC	CCCCGTCTCTGTATTCAACC

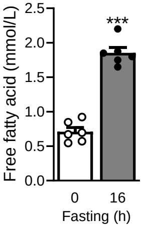

Figure S1. Fasting-Induced Lipolysis in Mice

Related to Figure 1.

Plasma FFAs measured in C57BL/6J mice before or after 16 hours fasting. All mice were male and fed a normal diet. Values are presented as mean ± SE. (n = 6). Significant differences were determined by Student’s t-test compared to baseline (0 hour) : ***$P < 0.001$.

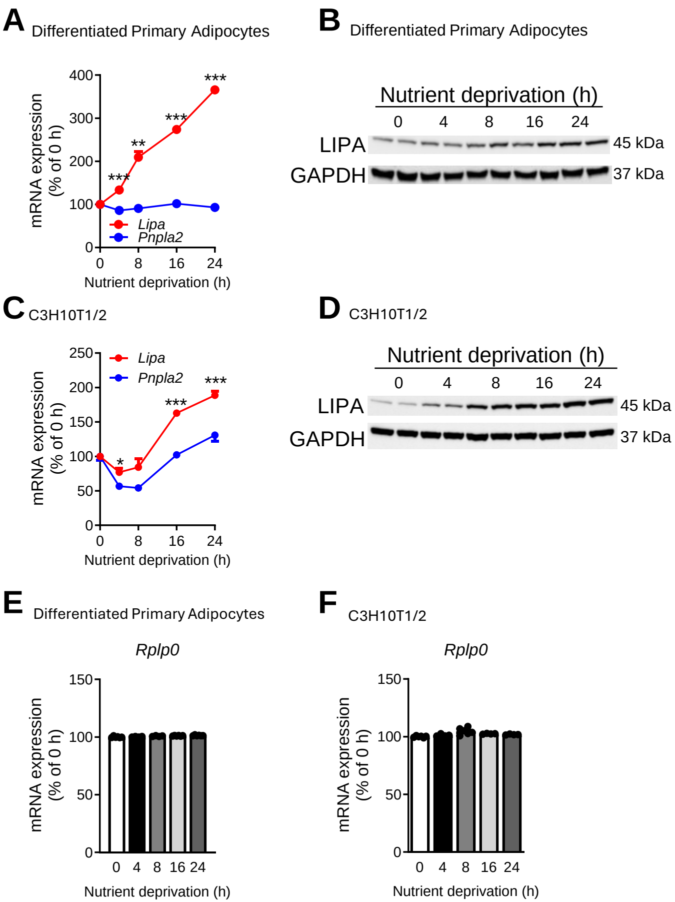

Figure S2. Nutrient Deprivation Induces Lipa Expression without Affecting Housekeep Gene Expression

Related to Figure 1.

(**A**) Gene expression of *Lipa* and ATGL (*Pnpla2*) (n = 4-6) and (**B**) protein expression of LIPA in differentiated primary adipocytes derived from iWAT SV cells starved in EBSS containing 1 g/L glucose and 2% fatty acid-free BSA for indicated durations.

(**C**) Gene expression of *Lipa* and ATGL (*Pnpla2*) (n = 4-6) and (**D**) protein expression of LIPA in C3H1T1/2 adipocytes starved in EBSS containing 1 g/L glucose and 2% fatty acid-free BSA for indicated durations.

(**E**) Gene expression of 36B4 (*Rplp0*) in primary adipocytes and (**F**) C3H1T1/2 adipocytes.

Values are presented as mean ± SE. Significant differences were determined by Student's t-test compared to baseline (0 hour) : **P* < 0.05, **P < 0.01, ***P < 0.001.

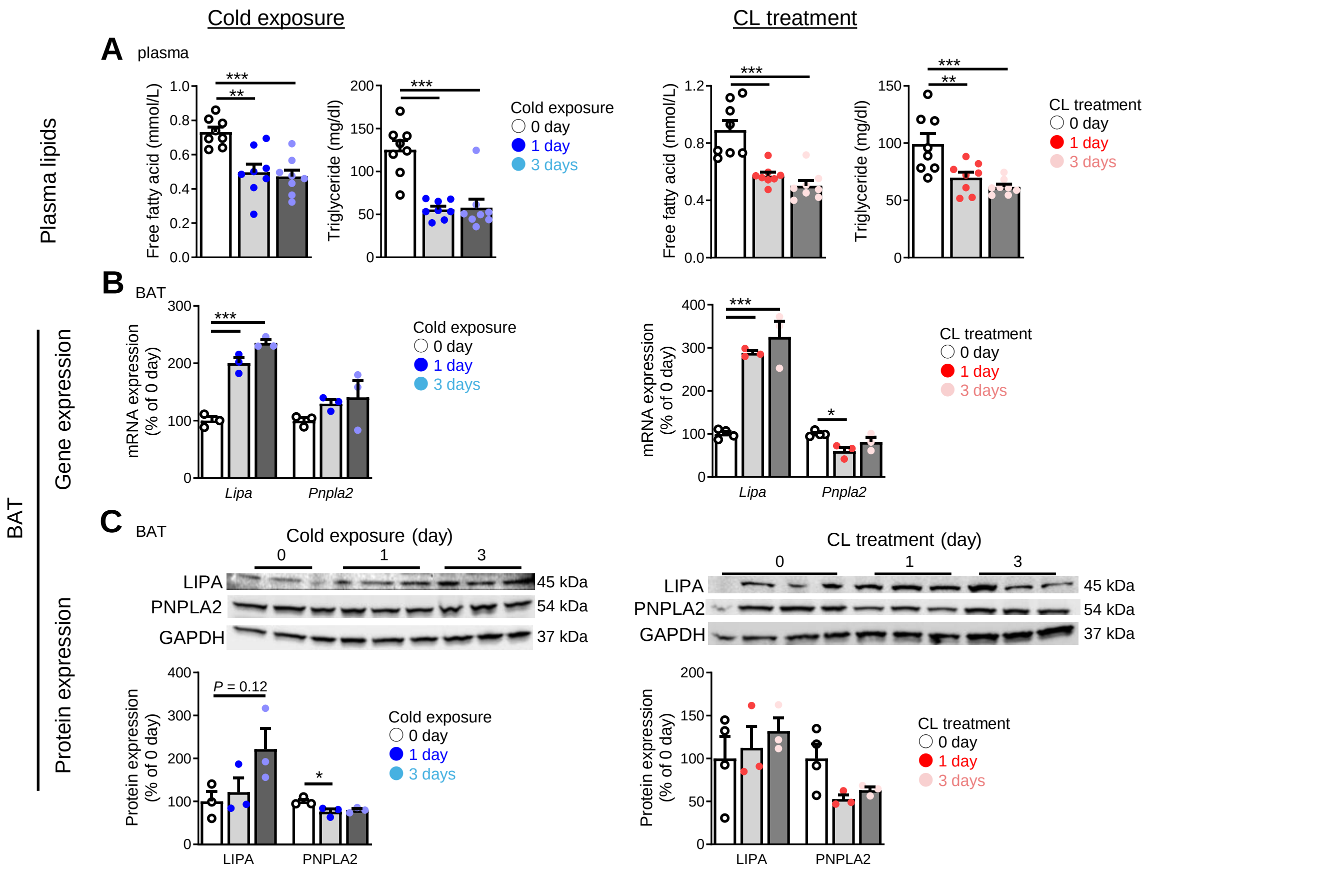

Figure S3. Plasma Lipid Levels and BAT Gene and Protein Expression in Response to Cold and β-Agonism

Related to Figure 1.

(**A**) Plasma FFAs and TGs in C57BL/6J mice exposed to cold (left panel) or treated with CL316,243 (CL) (right panel) for 0, 1, or 3 days (n = 8).

(**B**) Gene expression and (**C**) protein expression of LIPA and ATGL (PNPLA2) in interscapular brown adipose tissue (BAT) of C57BL/6J mice exposed to cold (left panel) or treated with CL (right panel) for 0, 1, or 3 days (n = 3-4).

All mice were male and fed a normal diet. Values are presented as mean ± SE. Significant differences were determined by one-way ANOVA with a post-hoc Tukey's HSD for comparisons with the indicated groups (*$P < 0.05$, **$P < 0.01$, ***$P < 0.001$) .

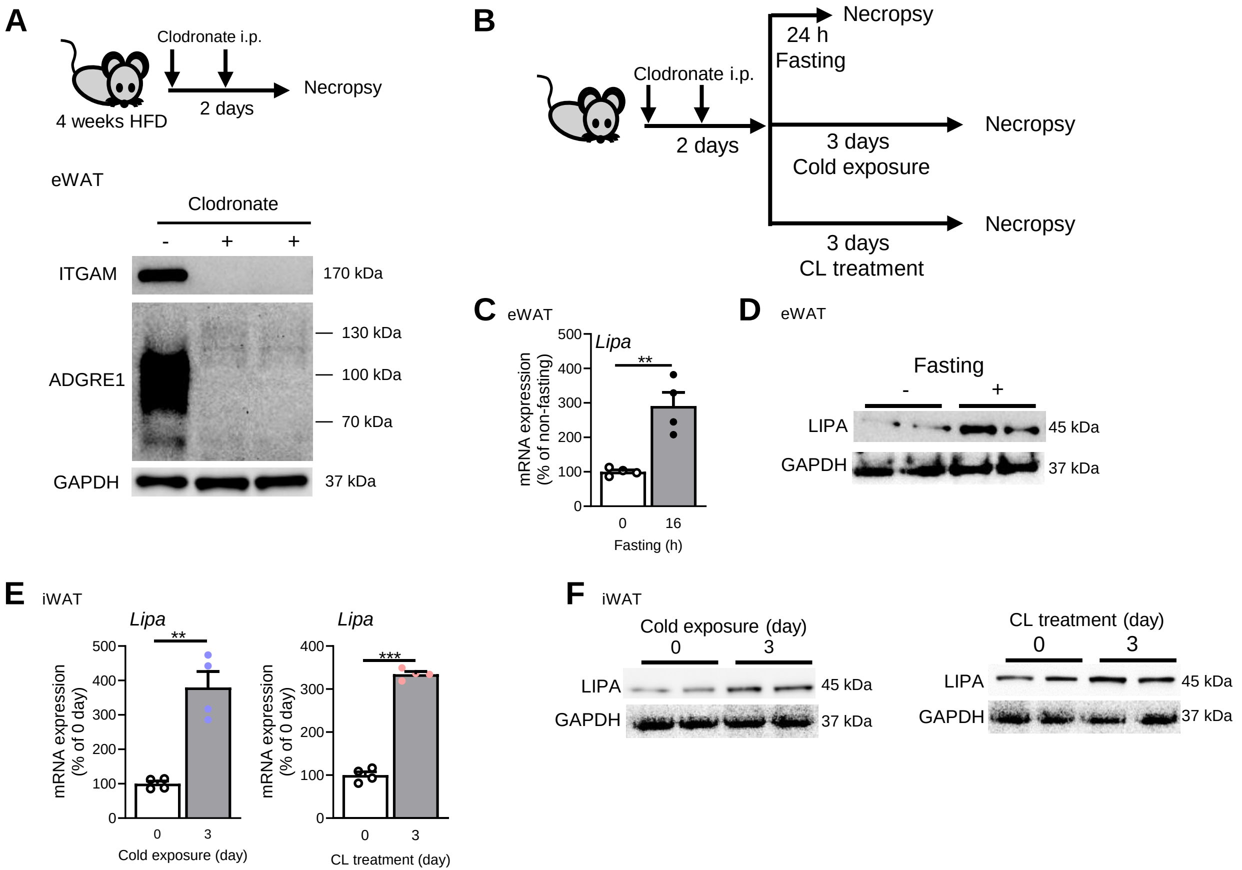

Figure S4. Macrophage Depletion Does Not Affect Lipolytic Stress-Induced Lipa Expression in Adipose Tissue

Related to Figure 1.

(**A**) Protein expression of CD11b (ITGAM) and F4/80 (ADGRE1) in the eWAT of 4-week high-fat diet (HFD)-fed mice intraperitoneally injected daily with 0.6 mg/mouse clodronate for 2 days.

(**B**) Schematic illustration of macrophage depletion in fasting, cold exposure, and CL316,243 (CL) treatment experiments.

(**C**) Gene expression of *Lipa* and (**D**) protein expression of LIPA in eWAT from mice fasted 16 hours (n = 4).

(**E**) Gene expression of *Lipa* and (**F**) protein expression of LIPA in iWAT from mice housed at 4ºC (left panel) or treated with CL (right panel) for 3 days (n = 4).

All mice were male and fed a normal diet. Values are presented as mean ± SE. Significant differences were determined by Student's t-test compared to untreated groups (0 day) ： $^{**}P < 0.01$, $^{***}P < 0.001$.

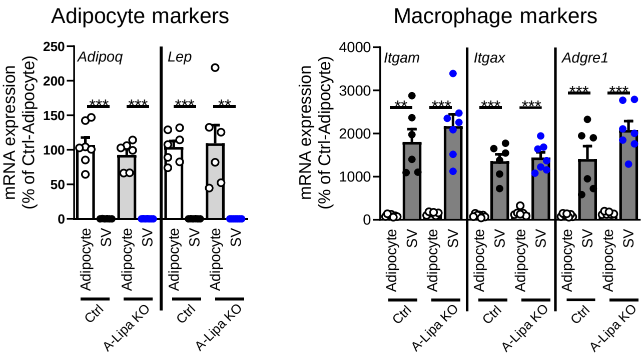

Figure S5. Markers of Adipocytes and Macrophages in Isolated Adipocyte and Stromal Vascular (SV) Fractions

Related to Figure 2.

Adipoq and *Lep* gene, CD11b (*Itgam*), CD11c (*Itgax*), and F4/80 (*Adgre1*) expression levels in eWAT separated by centrifugation into floating adipocyte and pelleted stromal vascular (SV) fractions from Ctrl (n = 6) and A-Lipa KO (n = 7) mice.

All mice were male and fed a normal chow diet. Values are presented as mean ± SE. Significant differences were determined by Student's t-test compared with indicated groups：**P < 0.01, ***P < 0.001.

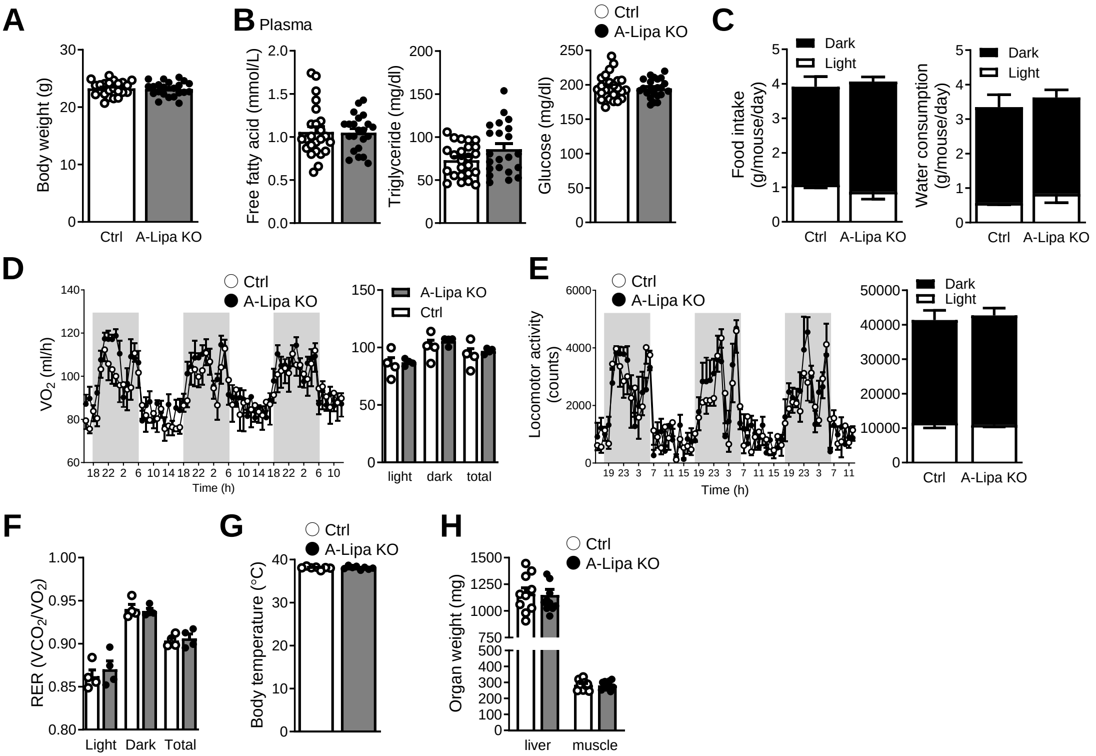

Figure S6. Adipose-Specific Lipa Knockout Mice Show No Basal Differences in Body Mass, Plasma Lipids, and Metabolic Characterization

Related to Figure 2.

(**A**) Body weight (n = 22) and (**B**) plasma FFA, TG, and glucose levels (n = 22).

(**C**) Food intake, water consumption (n = 4), (**D**) oxygen consumption (n = 4), (**E**) locomotor activity (n = 4), (**F**) respiratory exchange ratio (RER) (n = 4), (**G**) body temperature (n = 7-8), and (**H**) liver and muscle tissue weights (n = 11), in male mice.

All mice fed a normal diet and characterized at either 12 (metabolic cage measurements) or 16 weeks of age (tissue weights). Values are presented as mean ± SE.

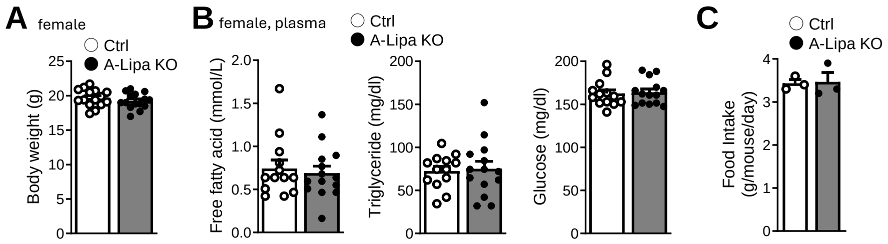

Figure S7. Baseline Metabolic Characterization of Female Adipose-Specific Lipa Knockout Mice

Related to Figure 2.

(**A**) Body weight, (**B**) plasma FFA, TG, and glucose levels, and (**C**) food intake measured in 16-week-old female A-Lipa KO (n = 14) and Ctrl (n = 13) mice. All mice fed a normal diet. Values are presented as mean ± SE.

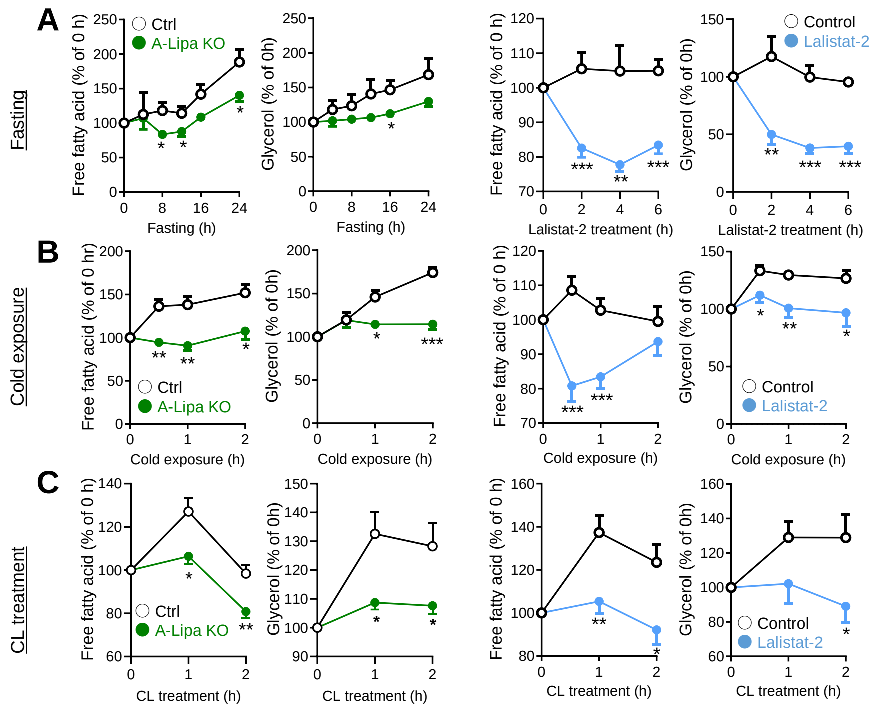

Figure S8. Relative Values of Free Fatty Acid and Glycerol in Fasting- and β-Agonist-Induced Adipocyte Lipolysis With or Without LIPA Disruption

Related to Figure 2

(**A**) Plasma FFA levels were measured in A-Lipa KO and Ctrl mice fasted 16 hours (n = 12) (left) or in C57BL/6J mice fasted for 16 hours and intraperitoneally injected with 30 mg/Kg body weight Lalistat-2 or vehicle control (n = 10-11) solution during followed by 4 hours fasting.

(**B**) Plasma FFA monitored in A-Lipa KO and Ctrl mice (n = 4) fasted and individually housed at 4 ℃ at indicated time points (left panel) or in C57BL/6J mice (n = 9-11) injected with 30 mg/Kg body weight Lalistat-2 or control solution one hour prior to indicated duration of individual housing at 4 ℃ without food (right panel).

(**C**) Plasma FFA (n = 4) measured at indicated time points in A-Lipa KO and Ctrl mice fasted for 16 hours then intraperitoneally injected with 1 mg/Kg body weight CL316,243 (CL). (left panel) or in C57BL/6J mice fasted for 16h, injected with 30 mg/Kg body weight Lalistat-2 (n = 9) or control solution (n = 8), and fasted for 90 more minutes prior to administration of 1 mg/Kg body weight CL without refeeding for indicated durations (right panel).

All mice were male and fed a normal chow diet. Values are presented as mean ± SE. Significant differences were determined by Student's t-test compared with Ctrl or Control group：*P < 0.05, **P < 0.01, ***P < 0.001.

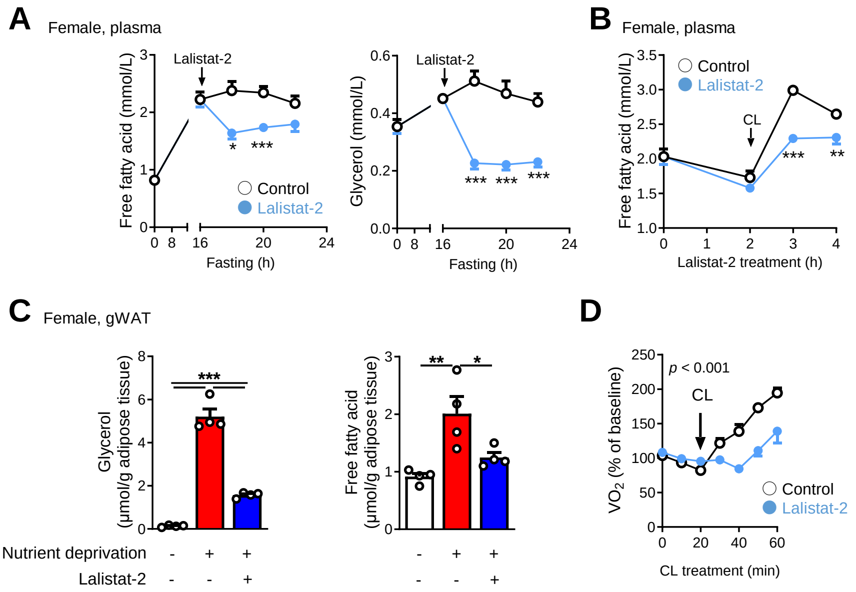

Figure S9. Lalistat-2 treatment Suppresses Fasting- and Cold-induced Lipolysis in Female C57BL/6J Mice

Related to Figure 2

(**A**) Plasma levels of FFA from female C57BL/6J mice fasted for 16 hours and intraperitoneally injected with 30 mg/Kg body weight Lalistat-2 (n = 7) or vehicle control solution (n = 8) followed by 4 hours of fasting.

(**B**) Plasma FFA levels collected at indicated time points (n = 8 or 7) in mice fasted for 16 hours, injected with 30 mg/Kg body weight Lalistat-2 or control solution, and fasted for 90 more minutes prior to administration of 1 mg/Kg body weight CL316,243 (CL) without refeeding for indicated durations.

(**C**) Glycerol and FFA levels in the supernatant of ex vivo cultured gonadal WAT (gWAT) from female C57BL/6J mice, starved in nutrient-free medium (EBSS buffer (1g/L glucose) with 2% fatty acid-free BSA) with or without 20 μM Lalistat-2 for indicated duration in hours (n = 4).

(**D**) Oxygen consumption (n = 4-5) in mice fasted for 16 hours, injected with 30 mg/Kg body weight Lalistat-2 or control solution, and fasted for 90 more minutes prior to administration of 1 mg/Kg body weight CL316,243 (CL) without refeeding for indicated durations.

All mice were female and fed a normal diet. Values are presented as mean ± SE. Significant differences were determined by Student's t-test (**A** and **B**) or by one-way ANOVA with a post-hoc Tukey's HSD test (**C** and **D**) for comparisons with the indicated or Control group (**P* < 0.05, ***P* < 0.01, ****P* < 0.001).

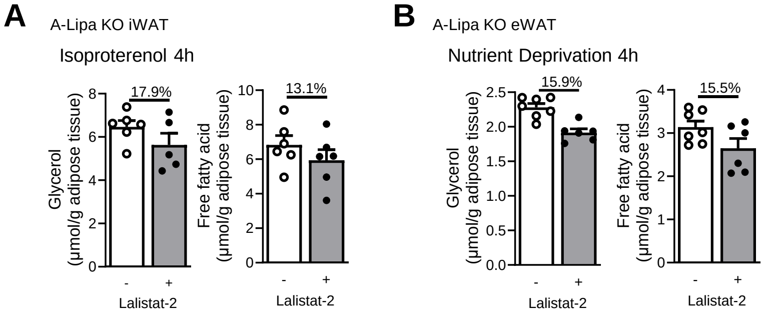

Figure S10. Free Fatty Acid and Glycerol Release Induced by Lipolytic Stress in WAT explants of A-Lipa KO mice Treated with Lalistat-2

Related to Figure 2.

(**A**) Supernatant glycerol and FFA levels from Lalistat-2- or vehicle-treated A-Lipa KO iWAT explants with 1 μM isoproterenol (iso) at 4 hours (n = 5-6).

(**B**) Supernatant glycerol and FFA levels from Lalistat-2- or vehicle-treated A-Lipa KO eWAT explants in nutrient-free medium (EBSS buffer (1g/L glucose) with 2% fatty acid-free BSA) (n = 6-7).

All mice were male and fed a normal chow diet. Values are presented as mean ± SE.

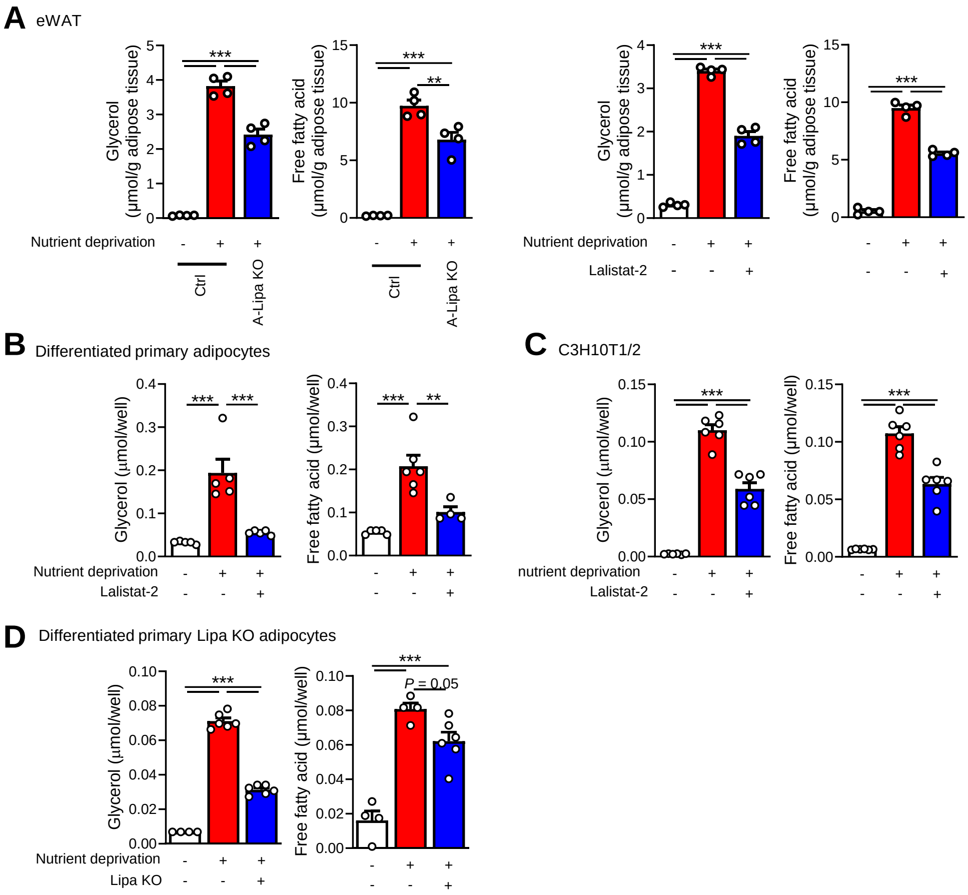

Figure S11. LIPA Disruption Suppressed Nutrient Deprivation-Induced Lipolysis

Related to Figure 3

(**A**) Under the condition of EBSS treatment (1g/L glucose) supplementing 2% fatty acid-free bovine serum albumin (BSA), supernatant glycerol and FFA levels from eWAT explants from A-Lipa KO versus Ctrl mice (left panel) or from 20 μM Lalistat-2- or vehicle control solution-treated eWAT explants from C57BL/6J mice (right panel), (**B**) primary adipocytes, and (**C**) C3H10T1/2 adipocytes, and (**D**) Lipa KO adipocytes.

Values are presented as mean ± SE. Significant differences were determined by one-way ANOVA with a post-hoc Tukey's HSD for comparisons with the indicated groups (*P < 0.05, **P < 0.01, ***P < 0.001).

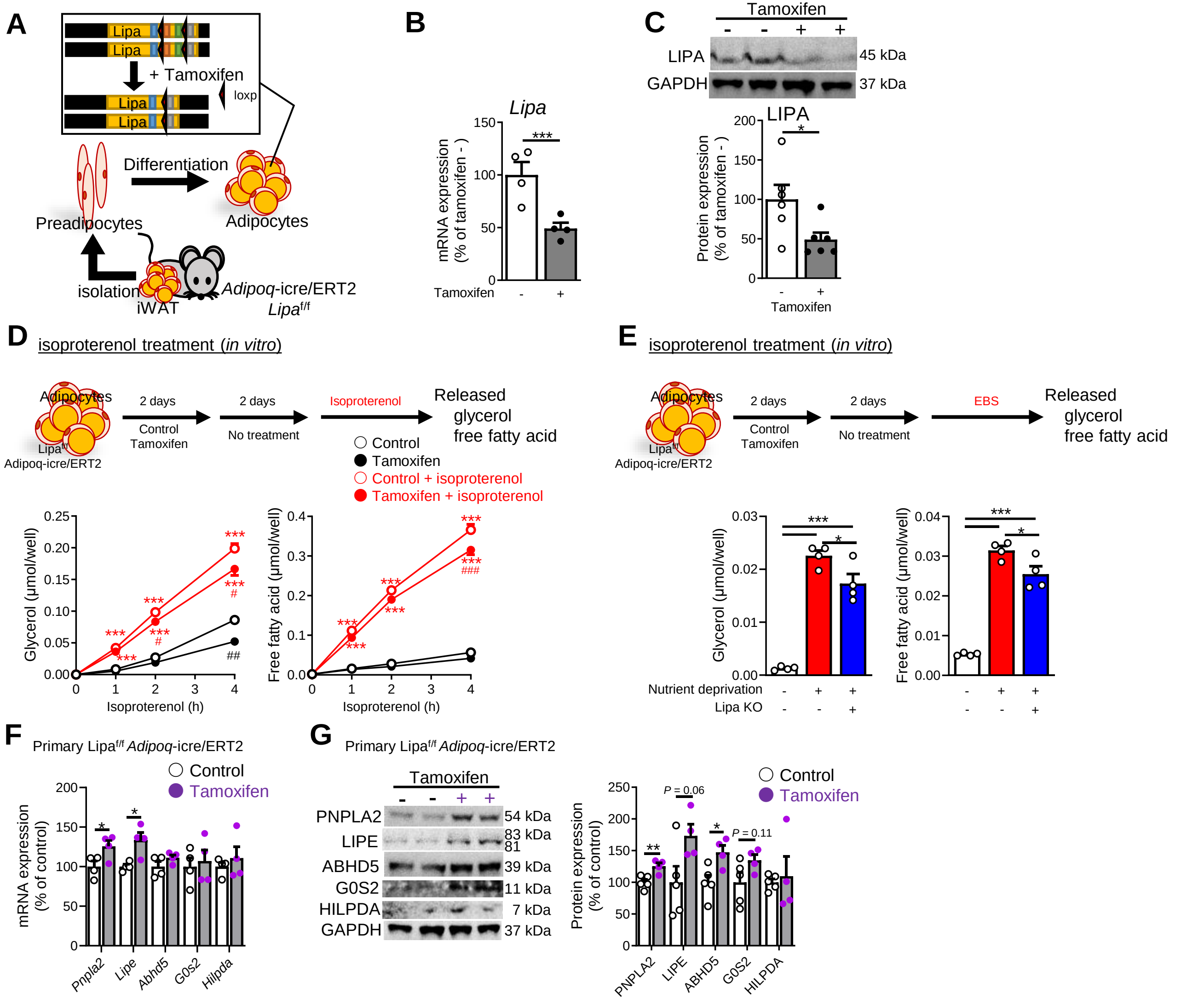
A
Lipa
+ Tamoxifen
loxp
Differentiation
Preadipocytes
Adipocytes
isolation
iWAT
Adipoq-icre/ERT2
Lipa f/f
B
Lipa
mRNA expression (% of tamoxifen -)
Tamoxifen
C
Tamoxifen
LIPA
GAPDH
45 kDa
37 kDa
Protein expression (% of tamoxifen -)
D
isoproterenol treatment (in vitro)
2 days
Control
Tamoxifen
No treatment
Isoproterenol
Released glycerol free fatty acid
Control
Tamoxifen
Control + isoproterenol
Tamoxifen + isoproterenol
Glycerol (μmol/well)
Free fatty acid (μmol/well)
Isoproterenol (h)
E
isoproterenol treatment (in vitro)
EBS
Nutrient deprivation
Lipa KO
F
Primary Lipa f/f Adipoq-icre/ERT2
mRNA expression (% of control)
Pnpla2
Lipe
Abhd5
G0s2
Hilpda
G
PNPLA2
LIPE
ABHD5
G0S2
HILPDA
54 kDa
83 kDa 81
39 kDa
11 kDa
7 kDa
Protein expression (% of control)
P = 0.06
P = 0.11

Figure S12. Tamoxifen-induced Lipa KO Suppresses Lipolysis Despite Compensatory Elevation of ATGL

Related to Figure 3.

(**A**) Schematic illustration of strategy to generate primary adipocytes with tamoxifen-induced LIPA knockout (TA-Lipa KO) through differentiating the SV cells isolated from the iWAT of *Adipoq*-iCre/ERT2 driven knockout of LIPA.

(**B**) Confirmation of LIPA depletion at mRNA (n = 4) and (**C**) protein level (n = 6) in TA-Lipa KO adipocytes with and without tamoxifen.

(**D**) Isoproterenol- or (**E**) EBSS-induced lipolysis measured as glycerol and FFA release in supernatants from TA-Lipa KO adipocytes (left panel) (n = 4) or primary adipocytes (right panel) (n = 3).

(**F**) Expression of mRNA and (**G**) protein of lipases, ATGL (PNPLA2) and HSL (LIPE), and ATGL-related cofactors, G0S2, HILPDA, and CGI-58 (ABHD5) in TA-Lipa KO adipocytes (n = 4-5).

All mice were male. Values are presented as mean ± SE. Significant differences were determined by Student's t-test (**B**, **C**, **F** and **G**) or by two- (**D**) or one- (**E**) way ANOVA with a post-hoc Tukey's HSD test for comparisons with the indicated groups (baseline groups: $^{*}P < 0.05$, $^{**}P < 0.01$, $^{***}P < 0.001$, or iso-treated groups : $^{\#}P < 0.05$, $^{\#\#}P < 0.01$, $^{\#\#\#}P < 0.001$).

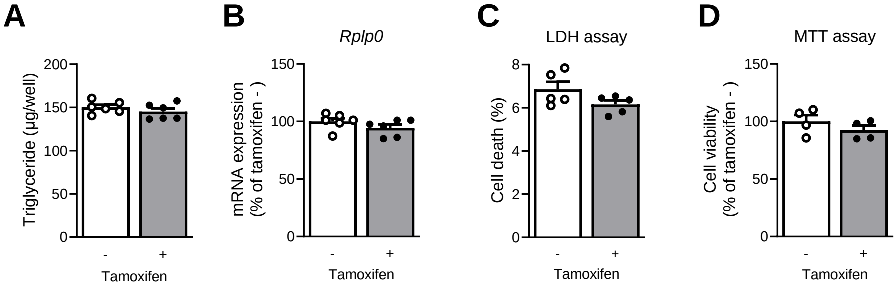

Figure S13. Effect of Tamoxifen Treatment on Primary Adipocytes

Related to Figure 3.

(**A**) TG accumulation (n = 6), (**B**) 36B4 (*Rplp0*) gene expression (n = 6), (**C**) LDH assay (n = 5), and (**D**) MTT assay (n = 4) were conducted in primary adipocyte in the absence or presence of tamoxifen.

Values are presented as mean ± SE. Significant differences were determined by Student's t-test compared with absence of tamoxifen.

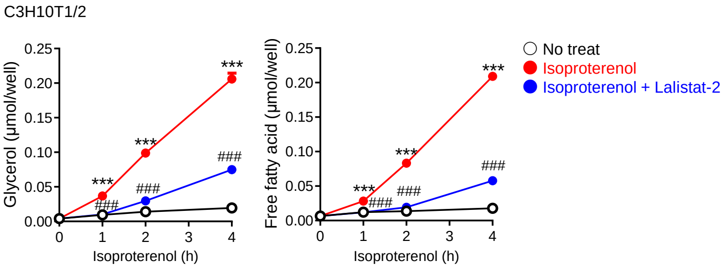

Figure S14. Lalistat-2 Treatment Suppressed Isoproterenol-Induced Lipolysis in C3H10T1/2 adipocytes

Related to Figure 3

Supernatant glycerol and FFA levels from C3H10T1/2 adipocytes with or without Lalistat-2 or isoproterenol treatment. Significant differences were determined by two-way ANOVA with a post-hoc Tukey's HSD for comparisons with the indicated groups (No treat: ***P < 0.001, or iso-treated groups : $^{###}P$ < 0.001).

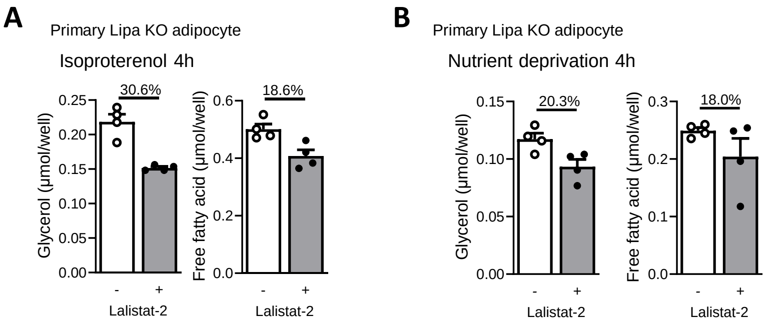

Figure S15. Free Fatty Acid and Glycerol Release Induced by Lipolytic Stress in Primary Adipocytes of A-Lipa KO mice Treated with Lalistat-2

Related to Figure 3.

(**A**) Supernatant glycerol and FFA levels from Lalistat-2- or vehicle-treated Lipa KO adipocytes with 1 μM isoproterenol (iso) at 4h (n = 4).

(**B**) Supernatant glycerol and FFA levels from Lalistat-2- or vehicle-treated Lipa KO adipocytes under the condition of EBSS treatment (1g/L glucose) supplementing 2% fatty acid-free BSA (n = 4).

Values are presented as mean ± SE.

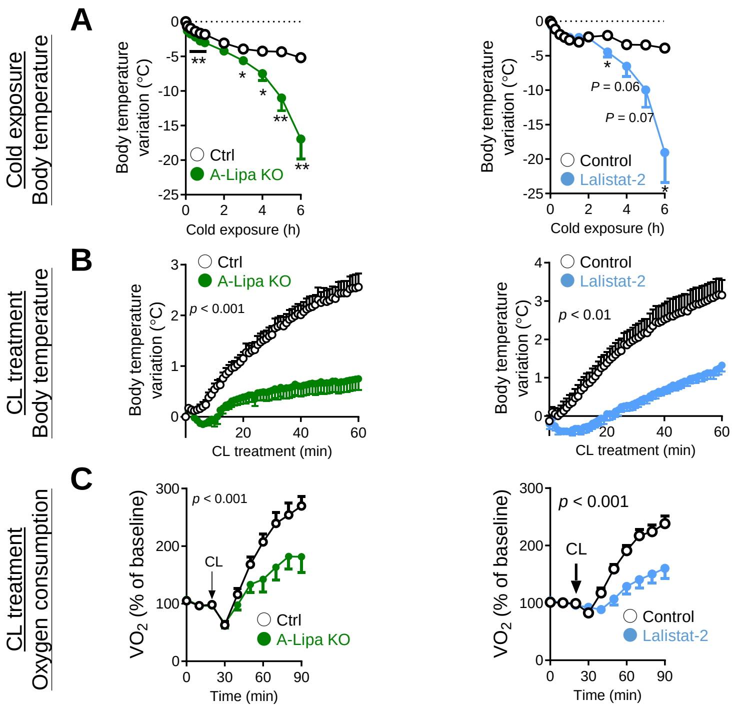

Figure S16. Absolute Values of Body Temperature and Oxygen Consumption Measurements in Setting of LIPA Deficiency Upon Cold or CL316,243 treatment

Related to Figure 4.

(**A**) Body temperatures monitored in A-Lipa KO and Ctrl mice (left panel) (n = 7-8) or in C57BL/6J mice (right panel) injected with 30 mg/Kg body weight Lalistat-2 or vehicle (n = 5-6) one hour prior to indicated duration of individual housing at 4 ℃ without food.

(**B**) Body temperature (n = 10) and (**C**) oxygen consumption (n = 6) measured at indicated time points in A-Lipa KO and Ctrl mice intraperitoneally injected with 1 mg/Kg body weight CL316,243 (CL) or in C57BL/6J mice injected with 30 mg/Kg body weight Lalistat-2 or control solution two hours prior to CL treatment.

All mice were male and fed a normal chow diet. Values are presented as mean ± SE. Significant differences were determined by two-way ANOVA (**B** and **C**) or Student's t-test compared with Ctrl or Control group：$*P < 0.05$, $**P < 0.01$.

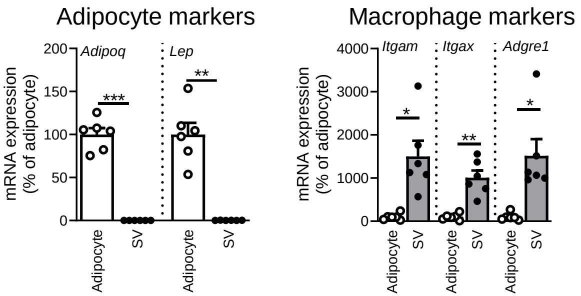

Figure S17. Markers of Adipocytes and Macrophages in Isolated Adipocyte and Stromal Vascular (SV) Fractions

Related to Figure 5.

Adipoq and *Lep* gene, CD11b (*Itgam*), CD11c (*Itgax*), and F4/80 (*Adgre1*) expression levels in eWAT separated by centrifugation into floating adipocyte and pelleted stromal vascular (SV) fractions from ND- and HFD-fed mice (n = 6).

All mice were male. Values are presented as mean ± SE. Significant differences were determined by Student's t-test compared with indicated groups： *P < 0.05, **P < 0.01, ***P < 0.001.

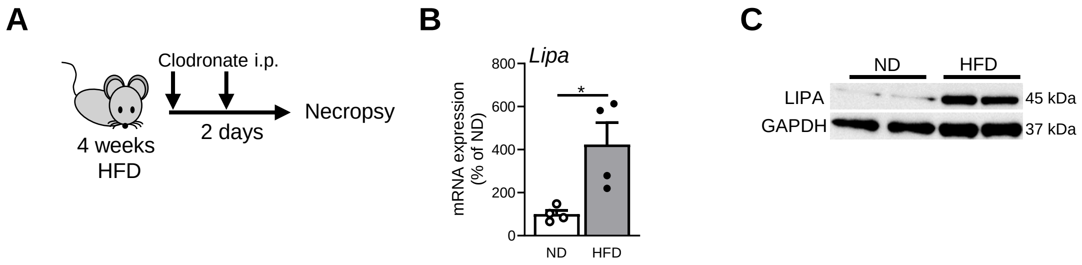

Figure S18. Macrophage Depletion Does Not Affect Adipose Tissue Lipa Expression in Setting of Diet-Induced Obesity

Related to Figure 5.

(**A**) Schematic illustration of macrophage depletion conducted by clodronate treatment in high-fat diet (HFD)-fed mice.

(**B**) Gene expression of *Lipa* and (**C**) protein expression of LIPA in eWAT from mice (n = 4).

All mice were male. Values are presented as mean ± SE. Significant differences were determined by Student's t-test compared to ND group : $*P < 0.05$.

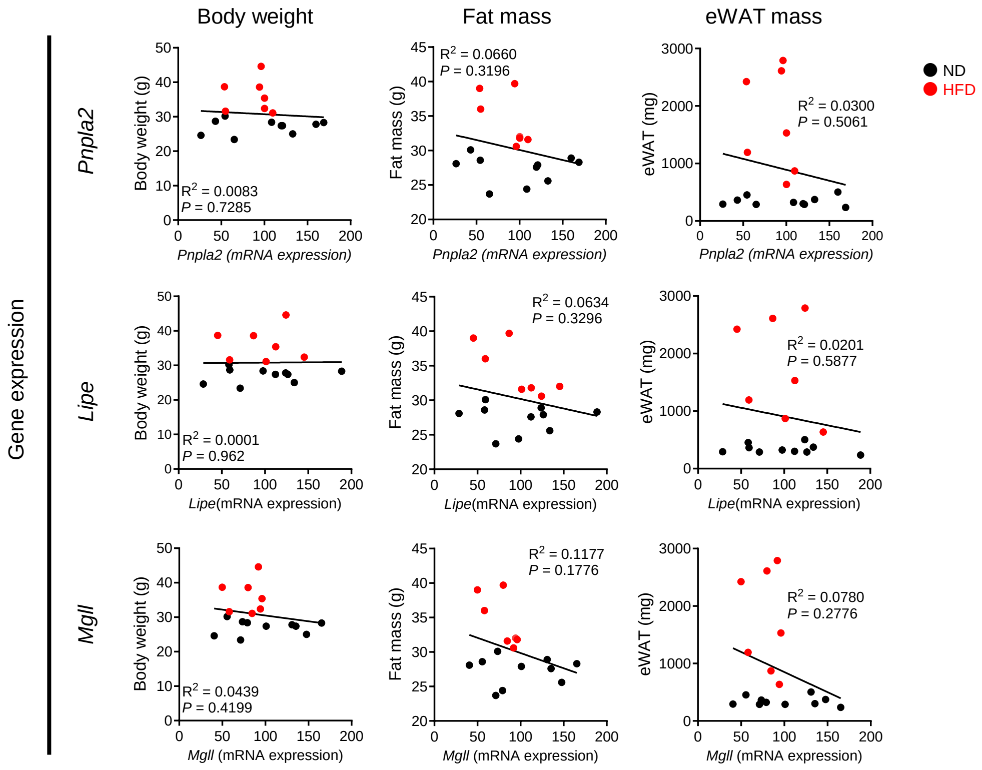

Figure S19. Cytoplasmic Lipase Gene Expression is Uncorrelated with Measures of Obesity

Related to Figure 5.

Matrix of Pairwise correlations of cytoplasmic lipases (ATGL (*Pnpla2*), HSL (*Lipe*), and MGL (*Mgll*) versus measures of obesity (body weight, total fat mass, and eWAT mass). All mice were male and fed a normal or high fat diet for 12 weeks initiated at 8 weeks of age prior to measurements. Values are presented as mean ± SE. The correlation (r square) and *P*-value were calculated by Pearson's r test.

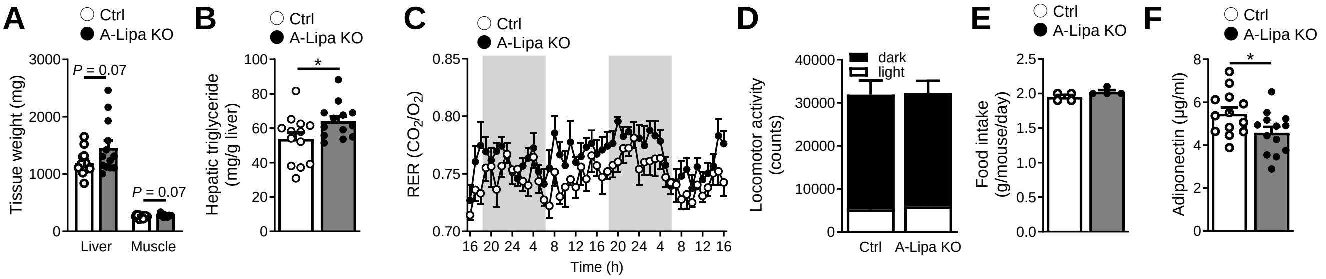

Figure S20. A-Lipa KO Mice Show an Increase in Hepatic TG Accumulation, and Lower Plasma Adiponectin due to Died-induced Obesity

Related to Figure 6

(**A**) Liver and muscle weight, (**B**) hepatic TG accumulation from Ctrl and A-Lipa KO mice after 16 weeks of HFD treatment (n = 13).

(**C**) Respiratory exchange ratio (n = 5), (**D**) locomotor activity (n = 5), and (**E**) food intake ((n = 4) in A-Lipa KO and Ctrl mice 10 weeks into the HFD study.

(**F**) Plasma adiponectin measured in A-Lipa KO and Ctrl mice fed HFD 16 weeks into the HFD study (n = 13).

All mice were male. Values are presented as mean ± SE. Significant differences were determined by Student's t-test compared with Ctrl groups：*P < 0.05, **P < 0.01.

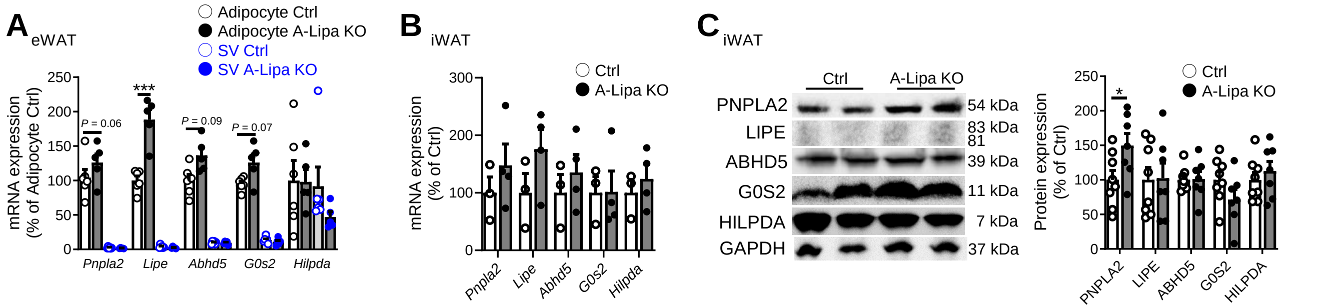

Figure S21. Lipolysis Contributed by LIPA is Independent of ATGL in iWAT

Related to Figure 7

(**A**) Gene expression in lipases, ATGL (*Pnpla2*) and HSL (*Lipe*), and ATGL-related cofactors, *G0s2*, *Hilpda*, and the CGI-58 (*Abhd5*) in homogenized eWAT separated by centrifugation into floating adipocyte and pelleted stromal vascular (SV) fractions from Ctrl and A-Lipa KO (n = 5-6) mice.

(**B**) Expression of mRNA (n = 3-4) and (**C**) protein (n = 7-8) in lipases, ATGL (PNPLA2) and HSL (LIPE), and ATGL-related cofactors, G0S2, HILPDA, and the CGI-58 (ABHD5) in iWAT from A-Lipa and Ctrl mice at 12 weeks old.

Values are presented as mean ± SE. Significant differences were determined by Student's t-test compared with indicated groups : *P < 0.05, ***P < 0.001

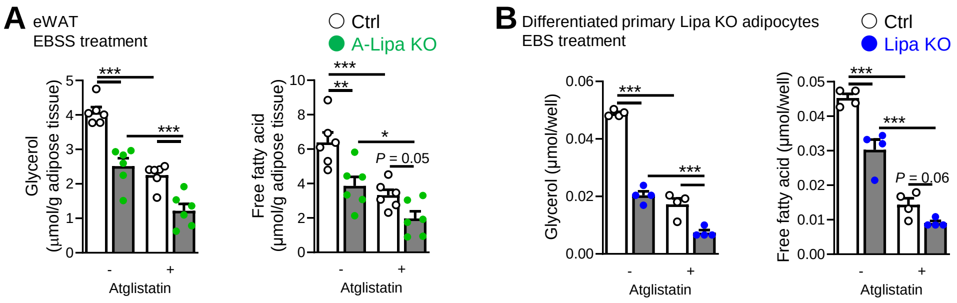

Figure S22. Nutrient Deprivation-Induced Lipolysis in WAT from A-Lipa KO and Lipa KO Adipocytes

Related to Figure 7

(**A**) Nutrient deprivation (EBSS buffer (1g/L glucose) with 2% fatty acid-free BSA)-induced supernatant glycerol and FFA levels from eWAT explants of A-Lipa KO mice and (**B**) Lipa KO adipocytes with or without Atglistatin.

Values are presented as mean ± SE. Significant differences were determined by one-way ANOVA with a post-hoc pairwise t-test for comparisons with the indicated groups (**P* < 0.05, ***P* < 0.01,****P* < 0.001).
