# Supplementary figures and images for: Identification of lysosomal lipolysis as an essential noncanonical mediator of adipocyte fasting and cold-induced lipolysis
Source: J Clin Invest. 2025 Mar 17;135(6):e185340. doi: 10.1172/JCI185340 (PMC11910232; doi:10.1172/JCI185340)

Figure 1.

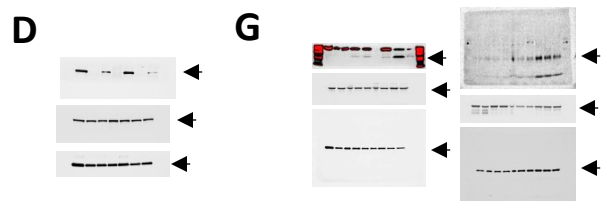

Figure 2.

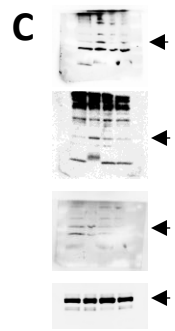

Figure 3.

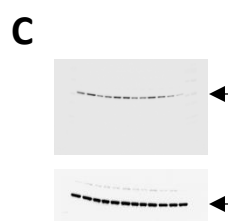

Figure 7.

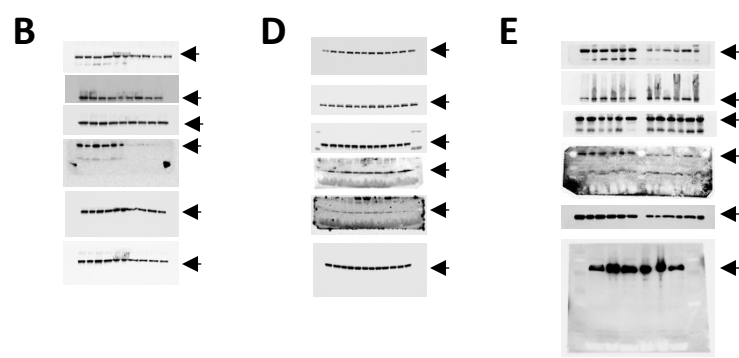

Figure S2.

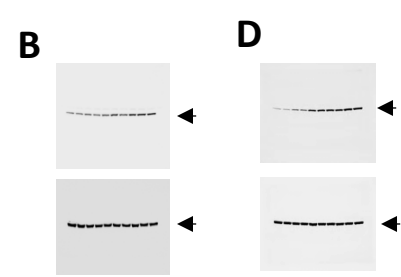

Figure S3.

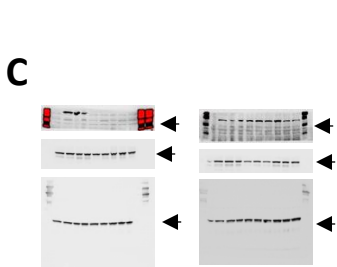

Figure S4.

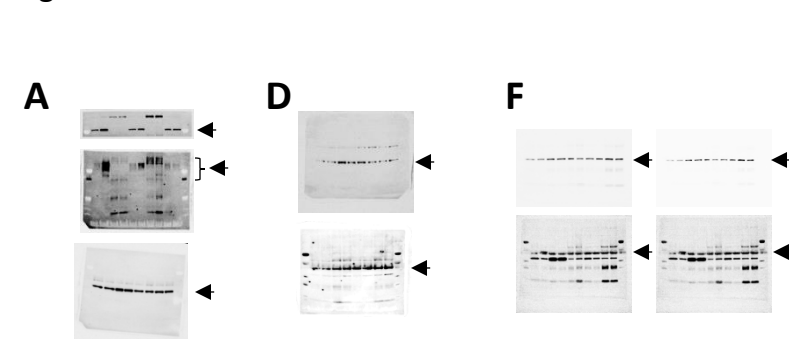

Figure S12.

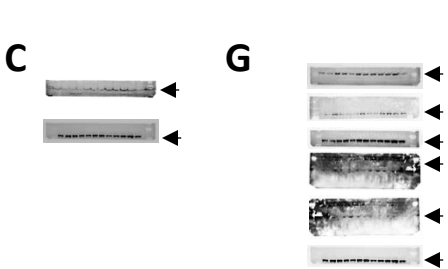

Figure S18.

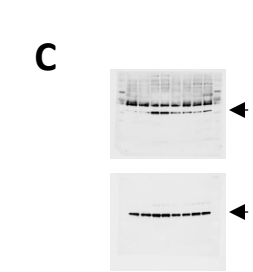

Figure S21.

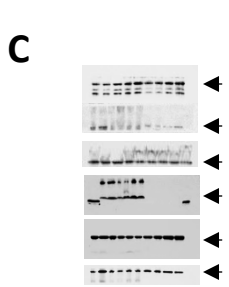

Supplement: Unedited blot and gel images [file jci-135-185340-s193.pdf]
